# *CNGB1*‐related rod‐cone dystrophy: A mutation review and update

**DOI:** 10.1002/humu.24205

**Published:** 2021-05-16

**Authors:** Marco Nassisi, Vasily M. Smirnov, Cyntia Solis Hernandez, Saddek Mohand‐Saïd, Christel Condroyer, Aline Antonio, Laura Kühlewein, Melanie Kempf, Susanne Kohl, Bernd Wissinger, Fadi Nasser, Sara D. Ragi, Nan‐Kai Wang, Janet R. Sparrow, Vivienne C. Greenstein, Stylianos Michalakis, Omar A. Mahroo, Rola Ba‐Abbad, Michel Michaelides, Andrew R. Webster, Simona Degli Esposti, Brooke Saffren, Jenina Capasso, Alex Levin, William W. Hauswirth, Claire‐Marie Dhaenens, Sabine Defoort‐Dhellemmes, Stephen H. Tsang, Eberhart Zrenner, Jose‐Alain Sahel, Simon M. Petersen‐Jones, Christina Zeitz, Isabelle Audo

**Affiliations:** ^1^ Sorbonne Université, Institut National de la Santé et de la Recherche Médicale, Centre National de la Recherche Scientifique Institut de la Vision Paris France; ^2^ Centre Hospitalier National d'Ophtalmologie des Quinze‐Vingts, INSERM‐DGOS CIC1423 Paris France; ^3^ Department of Clinical Sciences and Community Health University of Milan Milan Italy; ^4^ Ophthalmological Unit, Fondazione IRCCS Ca' Granda Ospedale Maggiore Policlinico Milan Italy; ^5^ Exploration de la vision et Neuro‐Ophthalmologie, CHU de Lille Lille France; ^6^ Faculté de Médecine Université de Lille Lille France; ^7^ University Eye Hospital, Centre for Ophthalmology University of Tübingen Tübingen Germany; ^8^ Institute for Ophthalmic Research, Centre for Ophthalmology University of Tübingen Tübingen Germany; ^9^ Department of Ophthalmology Columbia University, New York New York USA; ^10^ College of Medicine Chang Gung University Taoyuan Taiwan; ^11^ Department of Ophthalmology, Chang Gung Memorial Hospital Linkou Medical Center Taoyuan Taiwan; ^12^ Department of Ophthalmology, University Hospital LMU Munich Munich Germany; ^13^ Moorfields Eye Hospital London UK; ^14^ UCL Institute of Ophthalmology, University College London London UK; ^15^ Philadelphia College of Osteopathic Medicine Philadelphia Pennsylvania USA; ^16^ Wills Eye Hospital Philadelphia Pennsylvania USA; ^17^ Pediatric Ophthalmology and Ocular Genetics, Flaum Eye Institute, Pediatric Genetics, Golisano Children's Hospital University of Rochester Rochester New York USA; ^18^ Department of Ophthalmology University of Florida Gainesville Florida USA; ^19^ Univ. Lille, Inserm, CHU Lille, U1172‐LilNCog‐Lille Neuroscience & Cognition Lille France; ^20^ Jonas Children's Vision Care and Bernard & Shirlee Brown Glaucoma Laboratory New York New York USA; ^21^ Department of Pathology and Cell Biology Columbia University New York New York USA; ^22^ Stem Cell Initiative (CSCI), Institute of Human Nutrition, Vagelos College of Physicians and Surgeons New York New York USA; ^23^ Department of Ophthalmology The University of Pittsburgh School of Medicine Pittsburgh Pennsylvania USA; ^24^ Fondation Ophtalmologique Adolphe de Rothschild Paris France; ^25^ Department of Small Animal Clinical Sciences Michigan State University East Lansing Michigan USA; ^26^ University College London Institute of Ophthalmology London UK

**Keywords:** CNGB1, cyclic nucleotide‐gated channel, genotype‐phenotype correlation, inherited retinal disease, retinitis pigmentosa, rod‐cone dystrophy

## Abstract

Cyclic nucleotide‐gated channel β1 (*CNGB1*) encodes the 240‐kDa β subunit of the rod photoreceptor cyclic nucleotide‐gated ion channel. Disease‐causing sequence variants in *CNGB1* lead to autosomal recessive rod‐cone dystrophy/retinitis pigmentosa (RP). We herein present a comprehensive review and analysis of all previously reported *CNGB1* sequence variants, and add 22 novel variants, thereby enlarging the spectrum to 84 variants in total, including 24 missense variants (two of which may also affect splicing), 21 nonsense, 19 splicing defects (7 at noncanonical positions), 10 small deletions, 1 small insertion, 1 small insertion–deletion, 7 small duplications, and 1 gross deletion. According to the American College of Medical Genetics and Genomics classification criteria, 59 variants were considered pathogenic or likely pathogenic and 25 were variants of uncertain significance. In addition, we provide further phenotypic data from 34 *CNGB1*‐related RP cases, which, overall, are in line with previous findings suggesting that this form of RP has long‐term retention of useful central vision despite the early onset of night blindness, which is valuable for patient counseling, but also has implications for it being considered a priority target for gene therapy trials.

## 1 BACKGROUND

Inherited retinal diseases (IRDs) are a group of clinically and genetically heterogeneous disorders with an overall estimated prevalence that ranges between 1/3500 and 1/5000 worldwide. They are characterized by progressive photoreceptor degeneration (Hartong et al., 2006) with variable age of onset and degree of severity of vision loss. Up to now, more than 300 genes have been associated with IRDs (source: https://sph.uth.edu/retnet/) following all modes of inheritance, including Mendelian, mitochondrial, and rarely, digenism. Most of the proteins encoded by these genes are involved in cellular pathways that are crucial for photoreceptor or retinal pigment epithelium (RPE) homeostasis and functions (Wright et al., 2010). Retinitis pigmentosa (RP; MIM# 268000) or rod‐cone dystrophy (RCD) is the most common form of IRD. It affects approximately 1/4000 individuals and is characterized by rod dysfunction, followed by cone impairment (Hartong et al., 2006). Typical symptoms are night blindness, progressive visual field constriction and reduced visual acuity in later stages of the disease, resulting in some individuals being classified as “legally blind.” Over 80 different genes are associated with nonsyndromic RP (source: https://sph.uth.edu/retnet/). There is no current treatment for RP, however, there are several clinical trials underway (phase I–III), including various strategies (e.g., gene augmentation therapy, gene editing, antisense oligonucleotides, and others) showing promising results (Bainbridge et al., 2008, 2015; Cideciyan et al., 2013, 2019; MacLaren et al., 2014; Maguire et al., 2008, 2019; Schimmer & Breazzano, 2015).

Variants in *CNGB1* (MIM# 600724, Cyclic nucleotide‐gated channel β1) are associated with autosomal recessive RP 45 (RP45, MIM# 613767; Bareil et al., 2001), accounting for approximately 1%–4% of autosomal recessive RP cases (Ge et al., 2015; Maeda et al., 2008; Xu et al., 2014). Patients affected by *CNGB1*‐related RP usually experience the onset of night blindness during childhood. However, the constriction of the visual field becomes symptomatic much later and the visual acuity is usually well‐preserved during adulthood. Fundus abnormalities are typical of RP, with retinal vessel attenuation and mid‐peripheral “bone spicule” pigmentary clumping. Electroretinography shows a generalized dysfunction of the photoreceptors, predominantly affecting the rod system, with relative preservation of macular function in young adults (Hull et al., 2017).

C*NGB1* (MIM# 600724) encodes the 240‐kDa β subunit (CNGB1a) of the rod photoreceptor cyclic nucleotide‐gated (CNG) ion channel, containing a long cytosolic N‐terminus (glutamic acid‐rich protein [GARP]) domain and a channel domain, which includes a cyclic nucleotide‐binding domain (CNBD) (Ko¨rschen et al., 1995). This full‐length *CNGB1* is exclusively expressed in the retina, predominantly in the rod photoreceptors. However, shorter transcripts that encode the CNGB1b protein lacking the GARP domain are expressed in sensory neurons of the olfactory epithelium and form together with CNGA2 and CNGA4 subunits the native olfactory CNG channel (Kaupp & Seifert, 2002). In line with this, CNGB1 variants within the channel‐encoding part of the sequence result in peculiar olfactory symptoms. In particular, *CNGB1‐*mutant patients might experience an impaired sense of smell because the splice variant of the gene, CNGB1b, is expressed in the olfactory epithelium. The rod CNG channel consists of four subunits: one CNGB1a and three cyclic nucleotide‐gated channel α1 (CNGA1) (Kaupp & Seifert, 2002; Shuart et al., 2011). More specifically, CNGB1a subunits are important to ensure the correct localization of the channel in the plasma membrane of the outer segment of photoreceptors and help to modulate the channel activity (Biel et al., 2009). Under scotopic conditions, the CNBD of the CNG channels is occupied by cyclic GMP (cGMP), resulting in transient channel opening and a depolarizing inward current in rods. Upon light‐induced initiation of the phototransduction cascade, the intracellular cGMP concentration decreases through activation of the phosphodiesterase PDE6, which cleaves cGMP. This leads to the closure of the channels and hyperpolarization of the rods (Biel et al., 2009).

A mouse model of *CNGB1*‐retinopathy lacking exon 26 (*Cngb1*‐X26) is characterized by a slow progressive retinal degeneration with cell apoptosis and retinal gliosis (Huttl, 2005; Zhang et al., 2009). In this model, rods are the first to degenerate producing early (around 1 month of age) undetectable scotopic responses on the electroretinogram (ERG). Cone degeneration usually follows after 6 months of age and eventually these mice become blind after one year. In the inner retina, some morphological alterations were described in rod bipolar cells and horizontal cells (e.g., sprouting extensions, retraction of processes, and misplacement of cell bodies). More recently, Winkler et al. (2013) discovered a canine model of progressive retinal degeneration connected with a spontaneous variant in *Cngb1*, which closely resembled not only the *Cngb1*‐X26 mouse model but also the human RP45 phenotype. These studies using naturally occurring or generated animal models can provide further insights into the function of the protein and are important for preclinical studies in the development of therapeutic trials. For example, CNGB1 was at first considered a modulatory subunit of the CNG channels, and the retinal phenotype in *Cngb1*‐deficient mice and dogs was expected to be absent or milder than the one actually expressed. For this reason, it has been hypothesized that CNGB1 could be more than a modulatory subunit but it turned out to be required for assembly and targeting of native CNG channels to the outer segment, ensuring that only properly formed heteromeric channels reach the outer segment membrane. In fact, in *Cngb1*‐deficient mice only traces of the CNGA1 subunit are present at the membrane of outer segments of rods, confirming that homomeric CNGA1 channels are not efficiently trafficked and/or transported in the absence of CNGB1. The absence (or downregulation) of the CNG channels thus compromises rod function and eventually leads to the death of the photoreceptors through pathways that are still unclear. One possibility is the “equivalent light” hypothesis, which assumes that the absence of CNG channels is equivalent to the permanent closure of channels (as under a continuous bright light stimulation). Continuous exposure of experimental animals to light may result in photoreceptor degeneration through yet undefined mechanisms (Biel et al., 2009). Both *CNGB1*‐deficient mouse and canine models were used to attempt the rescue of the phenotype through gene augmentation therapy. This resulted in a robust, sustained restoration of rod function and retinal structural preservation in both models (Koch et al., 2012; Petersen‐Jones et al., 2018).

These preclinical successes raised the hope for applying *CNGB1* gene augmentation therapy to humans in the near future. However, before clinical trials, large cohort studies and natural history studies are warranted to ascertain whether there are suitable biomarkers of progression of retinal disease that can be used to assess the efficacy of therapy within a practical time frame. At the same time, the review and classification of the previously reported *CNGB1* variants in IRD cases could serve as a guide for the enrollment of patients in such trials.

## 2 MATERIAL AND METHODS

### 2.1 Literature search

A literature search was performed on Pubmed (updated on June 1st, 2020) to collect all reported variants on *CNGB1* (NM_001135639) in association with IRDs. Additional databases were queried, such as The Human Gene Mutation Database (HGMD® Professional 2017.4, last queried on September 1st, 2020), Leiden Open Variation Database (LOVD V.3.0, last queried on September 1st, 2020), and ClinVar (https://www.ncbi.nlm.nih.gov/clinvar/, last queried on September 1st, 2020). For each of the reported variants, we collected information on phenotype/genotype correlation, familial segregation, and documented minor allele frequency (MAF) as well as assessed functional impact through in silico pathogenic predictions tools (i.e., Mutation Taster [http://www.mutationtaster.org/ (Schwarz et al., 2014)], Sorting Intolerant From Tolerant SIFT [https://sift.bii.a-star.edu.sg/ (Sim et al., 2012)], Polymorphism Phenotyping v2 Polyphen‐2 [http://genetics.bwh.harvard.edu/pph2/ (Adzhubei et al., 2010)], Splice Site Prediction by Neural Network, NNSPLICE, [https://www.fruitfly.org/seq_tools/splice.html (Reese et al., 1997)]; Human Splicing Finder v.2.4.1, HSF, [http://www.umd.be/HSF/ (Desmet et al., 2009)] and evolutionary conservation (HomoloGene, available at https://www.ncbi.nlm.nih.gov/homologene and UCSC Genome Browser, available at https://genome.ucsc.edu/index.html). Guidelines from the American College of Medical Genetics and Genomics (ACMG) were used for variant classification (Richards et al., 2015).

### 2.2 Novel CNGB1 variants

In addition to the comprehensive literature search, we analyzed a multicenter large cohort of unrelated subjects with IRDs to find RP45 patients harboring known and novel *CNGB1* variants to prepare patients for future clinical trials. Each patient underwent a full ophthalmic examination by one of the investigators. Written informed consent was obtained from each subject. The study protocol adhered to the tenets of the Declaration of Helsinki and was approved by the local ethics committees. The affected index cases recruited at the Centre Hospitalier National d'Ophtalmologie des Quinze‐Vingts in Paris were genetically screened through targeted next generation sequencing (NGS) at the Institut de la Vision as previously described (Audo et al., 2012; Boulanger‐Scemama et al., 2015). The affected index cases recruited at the University Eye Hospital in Tübingen, Germany were genetically screened through targeted (NGS) at the Wissinger Lab, Institute for Ophthalmic Research in Tübingen as previously described (Glöckle et al., 2014; Weisschuh et al., 2016, 2018). The affected index cases recruited at the Department of Ophthalmology, Columbia University in New York, USA and in the Chang Gung Memorial Hospital in Taoyuan, Taiwan were genetically screened through whole exome sequencing as previously described (Tsang et al., 2014). The affected index case recruited at Moorfields Eye Hospital, London, UK was genetically tested through NGS at the Genomics Diagnostic Laboratory (Manchester Centre for Genomic Medicine, Manchester, UK). The affected index cases recruited at the Lille University Hospital, France, were genetically tested by NGS of a panel of 156 IRD genes in the genetics diagnostic laboratory (Department of Biochemistry and Molecular Biology, Lille, France). The data analysis was performed by a standard bioinformatics pipeline. The raw reads were filtered and aligned with the hg19 reference sequence from UCSC Genome Browser (https://genome.ucsc.edu/index.html) database (Kent et al., 2002). Variants were annotated using ANNOVAR (http://annovar.openbioinformatics.org/en/latest/) (Wang et al., 2010). The functional impact was assessed using in silico prediction tools described in the previous paragraph. Only rare variants with an MAF below or equal to 0.005 in the genome Aggregation Database (gnomAD, https://gnomad.broadinstitute.org/) were considered. All variants of interest were confirmed by Sanger sequencing, and segregation analyses were performed on the other family members when available.

### 2.3 Clinical investigation

A retrospective analysis was performed on the charts of the patients and their exams. Herein we reported the data from the last, most complete visit performed. When available, age of onset, best‐corrected visual acuity (BCVA), color vision, full‐field ERG (ff‐ERG), kinetic visual field, fundus examination, optical coherence tomography (OCT), and short‐wavelength fundus autofluorescence (SW‐FAF) data were collected.

### 2.4 CNGB1 VARIANTS

**Figure 1:**
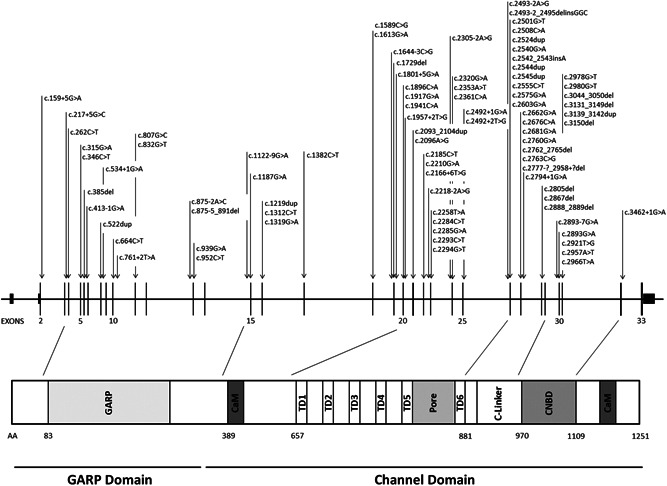
*CNGB1* variants underlying inherited retinal dystrophies. Nucleotide positions and translation correspond to CCDS45495.1 and NP_001288.3, respectively

**Figure 2:**
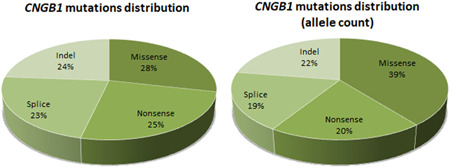
Pie charts showing the distribution of the *CNGB1* variants underlying inherited retinal diseases according to their types. On the left, the absolute distribution is shown. On the right, the distribution according to the allele count performed through the revision of the literature and the analysis of the cohort in this study is shown

A total of 62 genetic changes in *CNGB1* were found in the literature underlying IRD. In addition, we identified 22 novel changes in our cohort (Figure 1 and Table S1). Altogether, the sequence variant spectrum includes 24 missense variants (28%), 2 of which may also affect splicing, 21 nonsense (25%), 19 splicing defects (23%; 7 at noncanonical positions), and 20 changes in protein length and/or frameshift (24%; 10 small deletions, 1 small insertion, 1 small insertion–deletion, 7 small duplications, and 1 gross deletion) (Figure 2 and Table 1).

**Table 1:** List of *CNGB1* variants associated with inherited retinal dystrophies

Genomic start position (hg19)	Exon/intron	cDNA (NM_001297.5)	Protein change (NP_001288.3)	ACMG classification (criteria)	Phenotype	References	Frequency		
							Index Cases	Allele count	Hom Cases
58001027	IVS2	c.159+5G>A	p.?	Uncertain significance (PM2, PP3)	RCD	This study	1	1	0
57998386	IVS3	c.217+5G>C	p.?	Uncertain significance (PM2, PP3)	RCD	Oishi et al. (2014); This study	3	4	1
57998062	4	c.262C>T	p.(Gln88*)	Pathogenic (PVS1, PM2, PP1, PP3)	RCD	Ellingford et al. (2016); Afshar et al. (2019); Carss et al. (2017)	4	4	0
57996944	5	c.315G>A	p.(Trp105*)	Pathogenic (PVS1, PM2, PP3)	RCD	This study	1	2	1
57996913	5	c.346C>T	p.(Gln116*)	Pathogenic (PVS1, PM2, PP3)	RCD	Afshar et al. (2019); This study	2	2	0
57996773	6	c.385del	p.(Leu129Trpfs*148)	Pathogenic (PVS1, PM2, PP3)	RCD	Xiang et al. (2018)	1	2	1
57996515	IVS6	c.413‐1G>A	p.[=;Cys139Alafs*138]^a^	Pathogenic (PVS1,PM2, PS3, PP3, PP5)	RCD	Azam et al. (2011); Afshar et al. (2019); Charbel Issa et al. (2018)	6	11	5
57994756	8	c.522dup	p.(Lys175Glnfs*4)	Pathogenic (PVS1, PM2, PP1, PP3)	RCD	Lingao et al. (2016)	3	3	0
57994743	IVS8	c.534+1G>A	p.?	Pathogenic (PVS1, PM2, PP3)	RCD	Afshar et al. (2019)	1	1	0
57993889	10	c.664C>T	p.(Gln222*)	Pathogenic (PVS1, PM2, PP1, PP3)	RCD	Afshar et al. (2019); Carss et al. (2017)	2	2	0
57993790	IVS10	c.761+2T>A	p.?	Pathogenic (PVS1, PM2,PP3)	RCD	Hull et al. (2017)	1	2	1
57992344	11	c.807G>C	p.(Gln269His)	Likely pathogenic (PM1, PM2, PM3, PP3)	Isolated rod dysfunction	Afshar et al. (2019); Ba‐Abbad et al. (2019)	2	2	0
57992319	11	c.832G>T	p.(Glu278*)	Pathogenic (PVS1, PM2, PP3)	RCD	This study	1	1	0
57984446	IVS12	c.875‐2A>C	p.?	Pathogenic (PVS1, PM2,PP3)	RCD	Maeda et al. (2018)	1	2	1
57984428	IVS12	c.875‐5_891del	p.?	Pathogenic (PVS1, PM2, PP3)	RCD	Bernardis et al. (2016)	2	3	1
57984380	13	c.939G>A	p.(Trp313*)	Pathogenic (PVS1, PM2, PP1, PP3)	RCD	Fradin et al. (2016); This study	2	4	2
57984367	13	c.952C>T	p.(Gln318*)	Pathogenic (PVS1, PM2, PP1, PP3)	RCD	Afshar et al. (2019); Hull et al. (2017)	2	**2**	0
57974234	IVS14	c.1122‐9G>A	p.[=; Glu374Glufs*7]^b^	Pathogenic (PS3, PM2, PM3, PP1, PP3)	RCD	Petersen‐Jones et al. (2018)	1	1	0
57974160	15	c.1187G>A	p.(Arg396Gln)	Uncertain significance (BP4, PM2)	RCD	Comander et al. (2017)	1	1	0
57973487	16	c.1219dup	p.(Glu407Glyfs*12)	Pathogenic (PVS1, PM2,PP3)	RCD	Charbel Issa et al. (2018); This study	2	2	1
57973394	16	c.1312C>T	p.(Gln438*)	Pathogenic (PVS1, PM1, PM2)	RCD	Charbel Issa et al. (2018)	1	2	1
57973387	16	c.1319G>A	p.(Trp440*)	Pathogenic (PVS1, PM2, PP1, PP3)	RCD	This study	1	1	0
57965773	17	c.1382C>T	p.(Thr461Met)	Uncertain significance (BS1, PP3)	RCD	Ellingford et al. (2016)	1	1	0
57957231	18	c.1589C>G	p.(Pro530Arg)	Uncertain significance (PP1, PP3, BS1)	RCD	Fu et al. (2013)	1	2	1
57957207	18	c.1613G>A	p.(Trp538*)	Pathogenic (PVS1, PM2,PP3)	RCD	This study	1	2	1
57954451	IVS18	c.1644‐3C>G	p.?	Uncertain significance (PM2, PP3)	RCD	This study	1	2	1
57954363	19	c.1729del	p.(Glu577Serfs*6)	Pathogenic (PVS1, PM1, PM2)	RCD	Afshar et al. (2019)	1	2	1
57954286	IVS19	c.1801+5G>A	p.?	Uncertain significance (PM2, PP3)	RCD	Comander et al. (2017)	1	1	0
57953064	20	c.1896C>A	p.(Cys632*)	Pathogenic (PVS1, PM2, PP1)	RCD	Nishiguchi et al. (2013); Petersen‐Jones et al. (2018)	2	2	0
57953043	20	c.1917G>A	p.(Trp639*)	Pathogenic (PVS1, PM2, PM3)	RCD	Banerjee et al. (2017)	1	1	0
57953019	20	c.1941C>A	p.(Ser647Arg)	Uncertain significance (PM2, PP3)	RCD	This study	1	1	0
57953001	IVS20	c.1957+2T>G	p.?	Pathogenic (PVS1,PM2, PP3)	RCD	This study	1	1	0
57951242	21	c.2096A>G	p.(Asp699Gly)	Uncertain significance (PM2, PP3)	RCD	Ellingford et al. (2016)	1	1	0
57951234	21	c.2093_2104dup	p.(Cys698_Ile701dup)	Likely pathogenic (PM2, PM4, PP1, PP3)	RCD	Alshamrani et al. (2020)	1	1	0
57951166	IVS21	c.2166+6T>G	p.?	Uncertain significance (PM3,PP3)	RCD	This study	1	1	0
57950065	22	c.2185C>T	p.(Arg729*)	Pathogenic (PVS1, PM2,PP3)	RCD	Carss et al. (2017); Hull et al. (2017)	3	5	2
57950040	22	c.2210G>A	p.(Arg737His)	Uncertain significance (PM2, PP3)	RCD	Charbel Issa et al. (2018)	1	1	0
57949241	IVS22	c.2218‐2A>G	p.?	Pathogenic (PVS1, PM2, PP1, PP3)	RCD	Petersen‐Jones et al. (2018)	1	1	0
57949199	23	c.2258T>A	p.(Leu753*)	Pathogenic (PVS1, PM1, PM2)	Isolated rod dysfunction	Afshar et al. (2019); Ba‐Abbad et al. (2019)	2	2	0
57949173	23	c.2284C>T	p.(Arg762Cys)	Likely pathogenic (PM2, PM3, PP1, PP3, PP5)	RCD	Azam et al. (2011); Bocquet et al. (2013); Beryozkin et al. (2015); Consugar et al. (2015); Bernardis et al. (2016); Charbel Issa et al. (2018); Petersen‐Jones et al. (2018); This study	12	21	9
57949172	23	c.2285G>A	p.(Arg762His)	Uncertain significance (PM2, PP1, PP3)	RCD	Afshar et al. (2019); Carss et al. (2017)	2	4	2
57949164	23	c.2293C>T	p.(Arg765Cys)	Uncertain significance (PM2, PP1, PP3)	RCD	Schorderet et al. (2014); Habibi et al. (2016)	2	4	2
57949163	23	c.2294G>T	p.(Arg765Leu)	Uncertain significance (PM2, PP3)	RCD	Patel et al. (2016)	1	2	1
57946900	IVS23	c.2305‐2A>G	p.?	Pathogenic (PVS1, PM2, PP1)	RCD	This study	2	3	1
57912979	24	c.2320G>A	p.(Glu774Lys)	Uncertain significance (PM2, PP3)	RCD	This study	1	1	0
57946850	24	c.2353A>T	p.(Lys785*)	Pathogenic (PVS1, PM2, PP1)	RCD	This study	1	2	1
57946842	24	c.2361C>A	p.(Tyr787*)	Pathogenic (PVS1,PM2, PM3,PP3)	RCD	Xu et al. (2014); Banerjee et al. (2017)	2	2	0
57945656	IVS25	c.2492+1G>A	p.?	Pathogenic (PVS1, PM2, PP3)	RCD	Charbel Issa et al. (2018)	2	3	1
57911751	IVS25	c.2492+2T>G	p.?	Pathogenic (PVS1, PM2, PP3)	RCD	This study	1	1	0
57938777	IVS25	c.2493‐2_2495delinsGGC	p.?	Pathogenic (PVS1, PM2, PP1,PP3)	RCD	Maranhao et al. (2015)	1	2	1
57938781	IVS25	c.2493‐2A>G	p.?	Pathogenic (PVS1, PM2, PP1, PP3)	RCD	Maria et al. (2015)	1	1	0
57938771	26	c.2501G>T	p.(Arg834Leu)	Uncertain significance (PM2, PP3)	RCD	This study	1	1	0
57938764	26	c.2508C>A	p.(Tyr836*)	Pathogenic (PVS1, PM2,PP1,PP3)	RCD	Consugar et al. (2015); Petersen‐Jones et al. (2018)	2	2	0
57938748	26	c.2524dup	p.(Thr842Asnfs*10)	Likely pathogenic (PVS1, PM2)	RCD	Oishi et al. (2014)	1	2	1
57938732	26	c.2540G>A	p.(Gly847Glu)	Uncertain significance (PM2, PP3)	RCD	Afshar et al. (2019)	1	1	0
57938730	26	c.2542_2543insA	p.(Gly848Glufs*4)	Pathogenic (PVS1, PM2, PP3)	RCD	Charbel Issa et al. (2018)	1	2	1
57938728	26	c.2544dup	p.(Leu849Alafs*3)	Pathogenic (PVS1, PM2, PP3)	RCD	Kondo et al. (2004); Consugar et al. (2015); Ge et al. (2015; Ellingford et al. (2016); Afshar et al. (2019); Hull et al. (2017)	8	10	2
57938727	26	c.2545dup	p.(Leu849Profs*3)	Pathogenic (PVS1, PM2, PP3)	RCD	Petersen‐Jones et al. (2018)	1	1	0
57938717	26	c.2555C>T	p.(Pro852Leu)	Uncertain significance (PM2, PP3)	RCD	This study	1	2	1
57937858	26	c.2575G>A	p.(Val859Ile)	Uncertain significance (PM2, PP3)	RCD	This study	1	1	0
57938669	26	c.2603G>A	p.(Gly868Asp)	Uncertain significance (PM2, PP3)	RCD	Alshamrani et al. (2020); This study	2	3	1
57938697	26	c.2662G>A	p.(Ala888Thr)	Uncertain significance (PM2, PP3)	RCD	This study	1	1	0
57937844	27	c.2676C>A	p.(Tyr892*)	Pathogenic (PVS1, PM2, PP3)	RCD	Afshar et al. (2019); Carss et al. (2017)	2	2	0
57937839	27	c.2681G>A	p.(Arg894His)	Uncertain significance (PP1, PP3, BS1)	RCD	Ellingford et al. (2016)	1	1	0
57937760	27	c.2760G>A	p.(Trp920*)	Pathogenic (PVS1, PM2, PP3)	RCD	Beryozkin et al. (2015)	1	2	1
57937755	27	c.2762_2765del	p.(Tyr921Cysfs*15)	Pathogenic (PVS1, PM2, PP3)	RCD	de Castro‐Miró et al. (2016); This study	2	4	2
57937757	27	c.2763C>G	p.(Tyr921*)	Pathogenic (PVS1, PM2, PP3)	RCD	Charbel Issa et al. (2018)	1	1	0
57935256	28‐29	c.2777‐?_2958+?del	p.?	Pathogenic (PVS1, PM2, PP3)	RCD	Afshar et al. (2019)	1	2	1
57937725	IVS27	c.2794+1G>A	p.?	Pathogenic (PVS1, PM2, PP3)	RCD	This study	1	1	0
57935519	28	c.2805del	p.(Glu935Aspfs*2)	Pathogenic (PVS1, PM2, PP3)	RCD	Ge et al. (2015); This study	2	2	0
57935457	28	c.2867del	p.(Ile956Thrfs*15)	Pathogenic (PVS1, PM2, PP3)	RCD	This study	2	2	0
57935435	28	c.2888_2889del	p.(Phe963Serfs*4)	Pathogenic (PVS1, PM2, PP3)	RCD	Xu et al. (2014)	1	1	0
57935346	IVS28	c.2893‐7G>A	p.?	Uncertain significance (PM2, PM3, PP3)	RCD	This study	3	5	2
57935339	29	c.2893G>A	p.(Gly965Ser;?)	Likely pathogenic (PM1, PM2, PP1, PP3)	RCD	Ellingford et al. (2016)	1	1	0
57935311	29	c.2921T>G	p.(Met974Arg)	Uncertain significance (PM1, PM2, PP3)	RCD	Dan et al. (2020); This study	2	3	1
57935275	29	c.2957A>T	p.(Asn986Ile)	Likely pathogenic (PM1, PM2, PP3, PP5)	RCD	Simpson et al. (2011); Abu‐Safieh et al. (2013); Bernardis et al. (2016); Ellingford et al. (2016); Patel et al. (2016); Carss et al. (2017); Charbel Issa et al. (2018); Hull et al. (2017); Pérez‐Carro et al. (2018); Afshar et al. (2019); Fuster‐García et al. (2019); This study	24	34	10
57935266	29	c.2966T>A	p.(Val989Glu)	Likely pathogenic (PM1, PM2, PP1, PP3)	RCD	This study	1	2	1
57931817	30	c.2978G>T	p.(Gly993Val)	Likely pathogenic (PM1, PM2, PP1, PP3)	RCD	Bareil et al. (2001)	1	2	1
57931815	30	c.2980G>T	p.(Glu994*)	Pathogenic (PVS1, PM2, PP3)	RCD	Afshar et al. (2019); Carss et al. (2017)	2	2	0
57931745	30	c.3044_3050del	p.(Gly1015Valfs*4)	Pathogenic (PVS1, PM2, PP3)	RCD	Charbel Issa et al. (2018)	1	1	0
57931394	30	c.3131_3149del	p.(Ala1044Glyfs*13)	Pathogenic (PVS1, PM2, PP3)	RCD	This study	1	1	0
57931401	31	c.3139_3142dup	p.(Ala1048Glyfs*13)	Pathogenic (PVS1, PM2, PP3)	RCD	Carss et al. (2017); Charbel Issa et al. (2018)	3	3	0
57931393	31	c.3150del	p.(Phe1051Leufs*12)	Pathogenic (PVS1, PM2, PP3)	RCD	Nishiguchi et al. (2013); Ge et al. (2015); Petersen‐Jones et al. (2018); This study	5	7	2
57921758	IVS32	c.3462+1G>A	p.[=;Arg1081Argfs*68]^c^	Pathogenic (PVS1, PM2, PP3)	RCD	Kondo et al. (2004)	1	2	1
Total							170	236	67

*Note*: ACMG criteria for this study: PVS1: null variant (nonsense, frameshift, canonical ±1 or 2 splice sites or initiation codon); PS3: well‐established in vitro and​/or in vivo functional studies supportive of damaging effect on the gene or gene product; PM1: located on CNBD domain; PM2: frequency on gnomAD <0.5% and no homozygous cases (if not: BS1); PM3: variant detected in trans with a pathogenic variant; PM4: protein length changes as a result of in‐frame deletions/insertions in a nonrepeat region or stop‐loss variants; PP1: cosegregation with disease verified; PP3: at least 1 predictive algorithm suggest pathogenicity (for splice variants, score ≤−10%), if not: BP4; PP5: at least three previous publications report the variant as pathogenic; BP3: in‐frame deletions/insertions in a repetitive region without a known function; BP7: a synonymous variant for which splicing prediction algorithms predict no impact to the splice sequence nor the creation of a new splice site and the nucleotide is not highly conserved.

Abbreviations: ACMG, American College of Medical Genetics and Genomics; Hom, homozygous; RCD, rod‐cone dystrophy.

^a^ Effect validated by means of minigene assay by (Saqib et al., 2015).

^b^ Effect validated by means of in vitro assay by (Petersen‐Jones et al., 2018)

^c^ Effect validated by means of in vitro assay by (Becirovic et al., 2010).

The distribution of the different types of variants slightly changes when counting the alleles across all the reported cohorts; in fact, there is a higher prevalence of missense variants (39%) and a lower prevalence of nonsense (20%), splicing defects (19%), and indels (22%) (Figure 2 and Table 1). This is mostly related to the fact that the two missense variants c.2284C>T p.(Arg762Cys) (allele count: 21/236) and c.2957A>T p.(Asn986Ile) (allele count: 34/236) account together for the 23% of the investigated alleles. These variants were both found in different populations from different continents (Table 1).

According to the ACMG criteria, 59 variants were considered pathogenic or likely pathogenic and 25 variants were classified of uncertain significance (VUS; Table 1). All reported variants span the entire gene and therefore impact all functional domains (Figure 1). It is worth noticing that most of the variants occurring in the GARP domain of the protein are nonsense, frameshift, or affect splicing. Very few are the exceptions: c.1187G>A p.(Arg396Gln), c.1382C>T p.(Thr461Met), and c.807G>C p.(Gln269His). Although its frequency among the general population is as high as ~0.9% with 44 homozygous cases in gnomAD, variant c.1382C>T p.(Thr461Met) was considered VUS as Polyphen2 predicted it as “probably damaging.” On the contrary, variant c.1187G>A p.(Arg396Gln) was classified as VUS since its frequency among the general population is as low as 0.0046% but it was not predicted to be pathogenic by any of the applied algorithms, probably because of the low PhyloP (conservation) and Grantham (difference between amino acids) scores. Variant c.807G>C p.(Gln269His) is absent in the general population and is predicted as pathogenic by PolyPhen2 and SIFT. However, the associated phenotype was not the classical RP45, but an isolated rod dysfunction with only subtle peripheral pigmentary changes on fundus examination (Ba‐Abbad et al., 2019). In this case, the authors hypothesized a role of c.807G>C p.(Gln269His) in the sensitivity of the rod CNG channels to cGMP modulation: while the structural integrity of the channel might still allow the flow of Ca^2+^ ions to an extent that achieves nontoxic intracellular concentration, the variant might somehow affect the closure in response to light, abolishing the rod response. Overall, the interpretation of the missense variants on the GARP domain is still controversial as the precise function of the domain itself has not been established. However, its high conservation among vertebrates might suggest an important function. Of note, soluble GARP proteins are translated from the first 11–16 exons (Ardell et al., 2000) as separate cytosolic proteins that might take part in other cellular functions and interact with unknown partners (Ko¨rschen et al., 1995). Expression and proper outer segment localization of soluble GARP proteins is not affected in *Cngb1*‐X26 mice and dogs (Huttl, 2005; Winkler et al., 2013). To study the effect of GARP deletion another mouse model with a genetic modification in exon 1 of *Cngb1* (*Cngb1*‐X1 mice) was generated (DeRamus et al., 2017; Zhang et al., 2009). While *Cngb1*‐X1 mice showed similar functional defects as *Cngb1*‐X26 mice, they in addition showed a dramatically compromised rod outer segment morphology suggesting that soluble and channel‐attached GARP proteins are essential for rod disk morphogenesis and outer segment integrity (Zhang et al., 2009). Other studies suggested a functional role of GARP in the control of rod CNG channel activity by interacting with the CNBD (Michalakis et al., 2011). GARP was shown to act as a gatekeeper, which directly binds to the CNBD to dampen channel activity, thus, lowering background channel noise and increasing the fidelity of the phototransduction cascade. This inhibitory effect of GARP can be relieved by binding of cGMP to the CNBD of both rod CNG channel subunits (CNGA1 and CNGB1). The clinical relevance of this function of GARP became evident when analyzing *CNGB1* variants, which structurally impair the CNBD. For instance, c.2978G>T leads to a Gly to Val substitution within the CNBD and was shown to abolish channel function by impairing the cGMP‐dependent release of this autoinhibitory effect of GARP on the channel (Michalakis et al., 2011). Further biochemical analyses are required to clarify the impact of the GARP domain and its mutant forms on the overall activity of the CNG channels.

Most of the *CNGB1* missense variants associated with the RP45 phenotype have been located in the channel domain, with two “hot spots” at the level of the transmembrane domains and at the level of the CNBD, both crucial for the protein structure and function. In our cohort, we identified 32 families (34 patients) carrying biallelic *CNGB1* variants, 23 of which carried at least one novel variant (Table 2). Overall, we found 35 distinct variants, including 12 missense, 8 nonsense, 9 splice defects, 5 small deletions, and 1 small duplication. Recurrent variants were the known c.2957A>T (six families: two European, three North African, and one unknown origin) and the novel c.2893‐7G>A p.(?) (3 families: two European, one Turkish origin).

**Table 2:** Clinical and genetic characteristics of patients carrying novel likely disease‐associated *CNGB1* variants included in the study

ID	Geographic origin	*CNGB1* mutations (NM_001297.5)		Cosegregation	Symptoms at onset	Age at diagnosis (years)	Age at visit (years)	BCVA (Snellen)		ff‐ERG	Fundus	
								OD	OS		OD	OS
F5240	European	c.2575G>A,p.(Val859Ile)	c.2662G>A p.(Ala888Thr)	Unaffected mother (CIC09167) hetc.2575G>A; unaffected father(CIC09168) hetc.2662G>A	Night blindness during infancy	12	14	20/50	20/50	NA	Waxy optic disc, vessels narrowing, peripheral atrophy with bone spicules	
CIC09166												
OPH2286	European	c.1917G>A p.(Trp639*)	c.2492+2T>G p.(?)	NA	Night blindness during early infancy	16	18	20/32	20/32	Undetectable scotopic responses, residual photopic responses	Waxy disk and narrow vessels. Few bone spicules along the retinal vessels in mid‐periphery	
SRP541	European	c.2867del p.(Ile956Thrfs*15)	c.2957A>T p.(Asn986Ile)	NA	Night blindness during infancy	29	29	20/25	20/20	NA	Waxy disk, narrow vessels, central atrophy, few mid‐peripheral bone spicules	
ARRP278	European(?)	c.413‐1G>A p.(?)	c.413‐1G>A p.(?)	NA	Visual field constriction	27	30	20/32	20/25	Undetectable	Waxy disk, narrow vessels, mid‐peripheral bone spicules	
TW8999826^#^	Asian	c.217+5G>C, p.(?)	c.2903T>G, p.(Met968Arg)	NA	Night blindness since the age of 18	NA	30	20/20	20/20	Undetectable	Waxy disk, narrow vessels, mid‐peripheral bone spicules	
TW20024045^#^	Asian	c.217+5G>C, p.(?)	c.2903T>G, p.(Met968Arg)	NA	Night blindness childhood	NA	31	20/20	20/20	Undetectable scotopic responses, residual photopic responses	Waxy disk, narrow vessels, mid‐peripheral bone spicules	
SRP995	European	c.2867del p.(Ile956Thrfs*15)	c.2893‐7G>A p.(?)	NA	Night blindness during infancy	27	31	20/25	20/20	Undetectable scotopic responses, residual photopic responses	Waxy disk, narrow vessels, mid‐peripheral bone spicules	
MRN:6822243	Hispanic/Caucasian	c.159+5G>A p.?	c.1941C>A p.(Ser647Arg)	NA	Night blindness during infancy, photophobia, decreased peripheral vision	31	34	20/32	20/32	Scotopic rod specific ERG b‐wave amplitudes were extinguished in both eyes	Bony spicules located on the outer equator and there is slightly enlarged cupping in the left eye	
OPH3784	North Africa	c.2320G>A p.(Glu774Lys)	c.2957A>T p.(Asn986Ile)	NA	Night blindness during early infancy	35	36	20/20	20/25	Undetectable scotopic responses, residual photopic responses	Normal optic nerve head. Some vessel narrowing. Few bone spicules in mid peripheral. Hypopigmented retina	
ARRP400	Western Asia?	c.2555C>T p.(Pro852Leu)	c.2555C>T p.(Pro852Leu)	NA	Night blindness during infancy	34	36	20/20	20/20	Some residual scotopic responses, reduced photopic responses	Waxy disk, narrow vessels, ERM, few mid‐peripheral bone spicules	
F4300 CIC07722	North Africa	c.2957A>T p.(Asn986Ile)	c.2957A>T p.(Asn986Ile)	NA			39	20/25	20/32	NA	Waxy optic disc, vessels narrowing, peripheral atrophy with bone spicules	
SRP266	Unknown	c.2794+1G>A p.(?)	c.2957A>T p.(Asn986Ile)	NA	Night blindness at the age of 20	20	41	20/20	20/20	Some residual scotopic responses, reduced photopic responses	Mid‐peripheral bone spicules and atrophy	
ARRP349	Western Asia	c.2603G>A p.(Gly868Asp)	c.2603G>A p.(Gly868Asp)	NA	Night blindness during infancy	10	41	20/80	20/100	Undetectable	Waxy disk, narrow vessels, mid‐peripheral bone spicules	
F141 CIC00189	European	c.832G>T p.(Glu278*)	c.2805del p.(Glu935Aspfs*2)	NA	Night blindness during infancy	32	43	20/320	20/250	Undetectable	Waxy optic disc with peripapillary atrophy, vessels narrowing, peripheral atrophy with bone spicules and central atrophy with RPE mottling	
MEH28189	European	c.346C>T p.(Gln116*)	c.3139_3142dup p.(Ala1048Glyfs*13)	NA	Night blindness from age 7	27	44	20/30	20/40	NA	Peripheral bone spicule pigmentation and outer retinal atrophy	
F1107 CIC01530	European	c.1644‐3C>G p.?	c.1644‐3C>G p.?	Unaffected father (CIC03328) hetc.1644‐3C>G; unaffected mother (CIC03508) hetc.1644‐3C>G	Night blindness during infancy	19	45	20/63	20/32	Undetectable	Waxy optic disc, vessels narrowing, peripheral atrophy with bone spicules	
F3791 CIC06919	North Africa	c.2957A>T p.(Asn986Ile)	c.2957A>T p.(Asn986Ile)	NA	Night blindness during infancy	39	45	20/20	20/20	Undetectable scotopic responses, residual photopic responses	Waxy optic disc, vessels narrowing, peripheral atrophy with bone spicules	
ARRP386	Western Asia(?)	c.2893‐7G>A p.(?)	c.2893‐7G>A p.(?)	NA	Night blindness during infancy	23	49	20/20	20/20	Undetectable	Waxy disk, narrow vessels, ERM, mid‐peripheral bone spicules	
ARRP398	Western Asia?	c.2893‐7G>A p.(?)	c.2893‐7G>A p.(?)	NA	Night blindness since the age of 15–16	19	49	20/20	20/20	Undetectable	Waxy disk, narrow vessels, ERM, mid‐peripheral bone spicules	
OPH1710	European	c.2185C>T p.(Arg729*)	c.2957A>T p.(Asn986Ile)	NA	Night blindness during teen‐age	NA	50	20/25	20/20	Undetectable scotopic responses, residual photopic responses	Normal optic nerve head. Some vessel narrowing. Few bone spicules in nasal and inferior retina. Hypopigmented retina	
MRN:8759303	European	c.3131_3149del, p.(Ala1044Glyfs*13)	c.2166+6T>G, p.(?)	NA	Night blindness and peripheral vision loss since childhood	NA	50	20/100	20/32	NA	Waxy disk, narrow vessels, mid‐peripheral bone spicules, macular hole	Waxy disk, narrow vessels, mid‐peripheral bone spicules, ERM
F463 CIC00691*	European	c.1319G>A p.(Trp440*)	c.2305‐2A>G p.?	Unaffected sister (CIC00732) het c.1319G>A; unaffected sister (CIC00692) het c.2305‐2A>G	NA	NA	51	20/125	20/50	Undetectable	Waxy optic disc, vessels narrowing, peripheral atrophy with bone spicules	
F463 CIC02695*	European	c.1319G>A p.(Trp440*)	c.2305‐2A>G p.?	Unaffected sister (CIC00732) het c.1319G>A; unaffected sister (CIC00692) het c.2305‐2A>G	NA	NA	51	20/50	20/20	Undetectable	Waxy optic disc, vessels narrowing, peripheral atrophy with bone spicules	
F2070 CIC04317	European	c.1219dup p.Glu407Glyfs*12	c.1957+2T>G p.?	NA	Night blindness during infancy	45	51	20/20	20/32	Undetectable scotopic responses, residual photopic responses	Waxy optic disc, vessels narrowing, peripheral atrophy with bone spicules	
F5462 CIC09504	North Africa	c.2284C>Tp.(Arg762Cys)	c.2284C>Tp.(Arg762Cys)	NA	Night blindness during infancy	NA	52	20/125	20/800	Undetectable	Waxy optic disc, vessels narrowing, peripheral and central atrophy; bone spicules in the periphery	
F4053 CIC07355	European	c.2305‐2A>G p.?	c.2305‐2A>G p.?	NA	Night blindness during infancy	45	55	20/25	20/20	Undetectable scotopic responses, residual photopic responses	Waxy optic disc, vessels narrowing, peripheral atrophy with bone spicules	
F5830 CIC10130	European	c.939G>A p.(Trp313*)	c.939G>A p.(Trp313*)	NA	Night blindness during infancy	22	56	20/32	20/32	Undetectable scotopic responses, residual photopic responses	Waxy optic disc, vessels narrowing, peripheral atrophy with bone spicules	
ARRP396	Western Asia(?)	c.315G>A p.(Trp105*)	c.315G>A p.(Trp105*)	NA	Night blindness during infancy	NA	58	20/40	20/63	Undetectable	Pallor of the optic disk, narrow vessels, diffuse RPE atrophy with sparing of the macula, peripheral bone spicules	
F3038 CIC05823	North Africa	c.2762_2765del p.(Tyr921Cysfs*15)	c.2762_2765del p.(Tyr921Cysfs*15)	NA			64	Hands Motion	20/160	Undetectable	Waxy optic disc, vessels narrowing, peripheral and central atrophy; bone spicules in the periphery	
MRN:1203567687	Unknown	c.2353A>T, p.(Lys785*)	c.2353A>T, p.(Lys785*)	NA	Night blindness during infancy	58	72	No light perception	No light perception	NA	Diffuse chorioretinal atrophy with peripheral pigmentation	
MEH16550	Unknown	c.1613G>A, p.(Trp538*)	c.1613G>A, p.(Trp538*)	NA	Night blindness during infancy	NA	73	20/30	No light perception	NA	Waxy disk, narrow vessels, mid‐peripheral bone spicules	Impossible to perform
F4517 CIC08096	European	c.3150del p.(Phe1051Leufs*12)	c.217+5G>A p?	NA	Night blindness during infancy	20	77	20/100	20/125	Undetectable	Waxy optic disc, vessels narrowing, peripheral atrophy with bone spicules	
SRP480	Unknown	c.2957A>T p.(Asn986Ile)	c.2501G>T p.(Arg834Leu)	NA								
SRP629	Unknown	c.2966T>A p.(Val989Glu)	c.2966T>A p.(Val989Glu)	NA								

*Note*: * and ^#^: Patients are siblings.

Abbreviations: BCVA, best‐corrected visual acuity; ERM, epiretinal membrane; ff‐ERG, full‐field electroretinogram; het, heterozygous; NA, not available; OD, right eye; OS, left eye; RPE, retinal pigment epithelium.

Among the cohort, we identified 22 novel variants of which, according to the ACMG classification criteria, 12 were pathogenic or likely pathogenic while 10 were VUS (Table 1). The latter included six missense variants and four noncanonical splice variants. All missense variants were predicted as “deleterious” by at least two prediction algorithms; they exhibit very low frequency in the general population and the corresponding amino acids are highly conserved across different species (Tables S1 and S2). Furthermore, five of them are located in the channel domain, at the level of the mutational “hot spot” previously mentioned: variants c.2320G>A p.(Glu774Lys), c.2501G>T p.(Arg834Leu), c.2555C>T p.(Pro852Leu), c.2575G>A p.(Val859Ile), and c.2662G>A p.(Ala888Thr) are in the transmembrane regions of the protein and very close to the channel pore. Variant c.1941C>A p.(Ser647Arg) is located between the calmodulin‐binding site and the transmembrane domains. A change in polarity given from the substitution of the neutral serine with an arginine (positively charged) might have an important impact on the activity and/or the structure of this region. However, further investigations are warranted to confirm the pathogenicity of these variants. The four noncanonical splice variants classified as VUS were c.159+5G>A, c.1644‐3C>G, c.2166+6T>G, and c.2893‐7G>A. All four variants have very low frequency in the general population, they were predicted as pathogenic by in silico analysis and the corresponding nucleotides were highly conserved among species (Tables S2–S4). Variants c.159+5G>A and c.2166+6T>G are predicted to shift the closest donor sites from their physiologic positions to c.159+7G and c.2166+12G, respectively. On the other hand, variants c.1644‐3C>G and c.2893‐7G>A are predicted to shift the closest physiologic acceptor sites in positions c.1644‐2A and c.2893‐5G, respectively. While this is highly suggestive for a pathogenic effect of these variants, further functional tests are needed for confirmation.

## 3 PREVALENCE DATA

The screening of the cohorts of IRDs from the different centers involved in this study revealed that biallelic variants of *CNGB1* would account for a prevalence ranging between 0.42% and 0.78%. In particular, a prevalence of 0.78% was found at the Centre Hospitalier National d'Ophtalmologie des Quinze‐Vingts in Paris, France (12 index subjects out of 1533 screened); a prevalence of 0.42% was found at the University Eye Hospital in Tübingen, Germany (9 index subjects out of 2156 screened); a prevalence of 0.46% was found at the Lille University Hospital in France (3 index subjects out of 649 screened); finally a prevalence of 0.63% was found at Moorfields Eye Hospital, London, UK (20 index subjects out of 3195 screened). These numbers are comparable with previous studies that reported a prevalence ranging from ∼0.4% to 1% among IRDs (Carss et al., 2017; Consugar et al., 2015; Habibi et al., 2016; Haer‐Wigman et al., 2017; Oishi et al., 2014; Pontikos et al., 2020).

## 4 CLINICAL CHARACTERISTICS OF PATIENTS CARRYING CNGB1 VARIANTS

There are limited published data on the *CNGB1* retinal phenotype, including all imaging modalities and functional assessment (Ba‐Abbad et al., 2019; Bareil et al., 2001; Fradin et al., 2016; Hull et al., 2017). The *CNGB1*‐related IRD phenotype is usually a young adult onset disease with slow progression and preserved visual acuity through late adulthood (Hull et al., 2017). However, most of the patients report the occurrence of night vision disturbances since childhood or infancy. As previously mentioned, only one case of *CNGB1*‐related isolated rod dysfunction (as evaluated on ff‐ERGs) has been described (Ba‐Abbad et al., 2019). This patient had slightly prolonged adaptation to dim lighting conditions and no retinal alterations except for a mild mid‐peripheral retinal pallor. To the best of our knowledge, our cohort is currently the largest cohort with *CNGB1‐*related RP ever reported (Tables 2 and S5).

**Figure 3:**
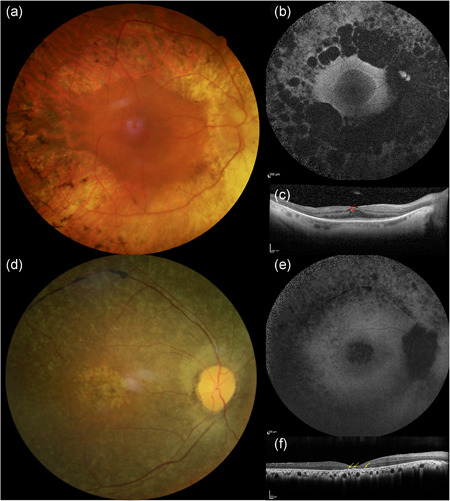
Clinical phenotype of a *CNGB1*‐related retinitis pigmentosa patient (F3791‐CIC06919). Fundus photographs of the right (a) and left (d) eyes show a waxy optic disc, narrow vessels and peripheral bone spicules. On short‐wavelength fundus autofluorescence (b, e), the central area of preserved tissue is surrounded by a ring of increased autofluorescence that demarks the limits of the peripheral atrophy. On optical coherence tomography (c, e) all retinal layers look centrally well preserved, while peripherally a thinning of the outer layers is evident

The 34 patients included had a mean age at the time of examination of 45.16 ± 14.67 years (median: 45 years; range 14–77 years; available data for 32 patients [*n*]). Age of diagnosis ranged between 10 and 58 (mean: 28.09 ± 11.81 years, median: 27; *n*: 21). All subjects experienced the onset of symptoms (primarily night blindness) during infancy or childhood. Visual acuity ranged from no light perception to 20/20 Snellen; however, most of the patients retained a visual acuity ≥20/40 Snellen in at least one eye (24/32 patients, 75%). Color vision was abnormal in at least one eye of 9/17 patients (52.94%), with 17 eyes showing dyschromatopsia, of which 9 with tritan (5 patients), 6 deutan (3 patient), and 2 tetartan (1 patient) defects. Visual fields were constricted in all patients, with a majority reduced to the 40 central degrees (vertical and horizontal diameters) or below in both eyes (V4 or III4 isopters; 13/20 patients, 65%). All patients had undetectable scotopic responses on ff‐ERGs. However, some residual cone responses were detectable in some of them (11 patients, 44%, *n*: 25), even in adult age. All subjects presented a classic form of RP with waxy optic disc pallor, attenuated retinal vessels, and peripheral bone spicules at fundus examination (Figure 2). The SW‐FAF showed the presence of a preserved central area of autofluorescence surrounded by a ring of hyperautofluorescence that clearly demarked the advancement of the peripheral outer retinal atrophy (Figures 2 and 3). This sign may represent an abnormal perifoveal accumulation of lipofuscin in the RPE due to increased outer segment dysgenesis (Lima et al., 2009; Robson et al., 2006) or a window defect caused by the atrophic alterations of the photoreceptors above a still intact RPE (Boulanger‐Scemama et al., 2019; Khateb, Nassisi, et al., 2019). In a recent study employing quantitative fundus autofluorescence, it was shown that SW‐AF in the ring reflects an actual increase in AF intensity within the ring relative to corresponding areas in the healthy retina. This finding is indicative of increased fluorophore production in impaired photoreceptor cells within the ring (Schuerch et al., 2017). This sign is usually associated with a preserved central vision (Khateb, Mohand‐Saïd, et al., 2019; Khateb, Nassisi, et al., 2019; Lima et al., 2012; Robson et al., 2003). Abnormal parafoveal rings of increased SW‐AF have been reported in approximately 59% of patients with RP (Murakami et al., 2008) although different genetic causes may have different prevalence (e.g., ~63% *PDE6A*, ~79% *PDE6B*, ~68% *MYO7A* (Khateb, Mohand‐Saïd, et al., 2019; Khateb, Nassisi, et al., 2019)). In our cohort, only 6/29 subjects did not show this para‐foveal feature (20.68%). All these cases showed an advanced atrophic involvement of all the posterior poles and very poor BCVA. Only in one case (F141‐CIC00189), there was evidence of central hypoautofluorescence with foveal involvement (BCVA: 20/320 and 20/250 Snellen in the right and left eye, respectively), even though the visual field was still well preserved (Figure 3). Consistently, this patient had central outer retinal thinning/atrophy on OCT unlike those with the normal foveal autofluorescence (Figure 3). A total of 10/29 patients (34.48%) had an epiretinal membrane (ERM) evident on OCT, although in most of the cases, it did not involve the center. Previous studies reported a prevalence of ERM that ranged between 0.6% and 64% in RP, depending on the OCT used and above all, not considering the genetic causes (Hagiwara et al., 2011; Khateb, Mohand‐Saïd, et al., 2019; Liew et al., 2019; Testa et al., 2014; Triolo et al., 2013). In our cohort, the prevalence of ERM is slightly higher than the 20% (2/10) previously reported by Hull et al. (2017) in *CNGB1*‐related RP. Other common OCT features were intraretinal hyperreflective foci (IHF) and intraretinal cysts (Figure 3). IHF may represent outer segment debris, RPE cell migration and/or phagocytes (Ho et al., 2011; Kuroda et al., 2014; Nagasaka et al., 2018). However, they are not specific to *CNGB1‐*related RP (Chen et al., 2016; Piri et al., 2015; Romano et al., 2019), neither of IRDs (Bolz et al., 2009; Christenbury et al., 2013; Matet et al., 2018; Nassisi et al., 2018; Ogino et al., 2012). In RCD, Kuroda et al. (2014) suggested that these hyper‐reflective dots could be associated with disease progression. However, the genetic analysis was not available in this study, therefore, the occurrence of IHF in correlation with specific gene defects in IRD remains to be established. Finally, intraretinal cysts were present bilaterally in 5/29 cases (17.24%), all of them without foveal involvement. This data is inferior to the previously reported prevalence of cystoid macular edema in RCD, which ranged among 26.9% and 58.6% (Adackapara et al., 2008; Hajali et al., 2008; Liew et al., 2019) but slightly higher than the 10% (1/10) reported in *CNGB1* patients (Hull et al., 2017). As the shorter isoform *CNGB1b* transcribed from the *CNGB1* locus is involved in the olfactory signal transduction, recent reports highlighted the presence of an impaired sense of smell in patients carrying biallelic *CNGB1* variants, in particular in the channel domain (as the GARP domain is not expressed in the olfactory cells) (Afshar et al., 2019; Charbel Issa et al., 2018). Given the retrospective nature of our study, we did not have any data regarding the sense of smell in our cohort, neither subjective nor objective. Overall, our study is consistent with the previous findings that suggest that *CNGB1*‐related RP has a good prognosis for central vision despite the early onset of night blindness. Even though the present cohort is the largest *CNGB1*‐related retinopathy cohort reported so far in the literature, we were not able to draw any specific conclusion on phenotype/genotype correlations, most likely due to the diversity of the sequence variant spectrum in a yet limited number of patients. However, coherently with previous reports, we did not see any direct correlation between different variants and phenotypic severity. For example, patients ARRP400 and F3791‐CIC06919, who harbor missense variants, had well preserved central vision and visual field into their third and fourth decades; however, SRP995, who also carries missense variants, showed an early onset disease with a significant reduction of BCVA and visual field. Conversely, among the four patients with relatively preserved visual fields (≥120° of field) in their 30s and 40s, there were two subjects (F141‐CIC00189 and SRP995) with null variants on both alleles.

**Figure 4:**
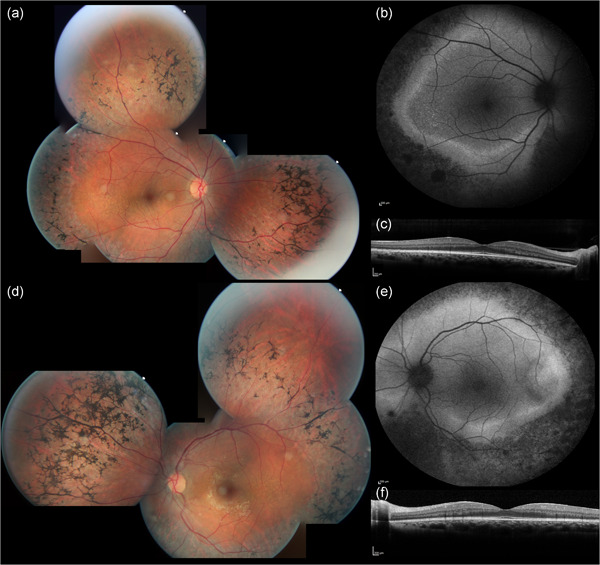
Clinical phenotype of two *CNGB1*‐related retinitis pigmentosa patients. Patient F1107‐CIC01530 (a) shows widespread peripheral RPE changes with bone spicules on fundus photographs (top row). These changes (black on the SW‐FAF [middle row]) surround an area with three different types of autofluorescence: a central normal autofluorescence (area of preserved tissue), a thin ring of intense hyperautofluorescence, and an outer ring of relatively preserved autofluorescence (outer retinal atrophy). These three areas are easy to recognize on OCT (bottom row) where there is also evidence of small intraretinal cysts (red arrows) and a small ERM. Patient F141‐CIC00189 (b) shows a central area of RPE and outer retina atrophy (top row), also evident on the SW‐FAF, which is surrounded by a preserved area of autofluorescence. In this patient, the demarcation between preserved and diseased tissue is not well demarked. OCT (bottom row) confirms the central atrophy and shows also the presence of intraretinal hyperreflective foci (yellow arrows) as well as an adherent ERM. ERM, epiretinal membrane; OCT, optical coherence tomography; RPE, retinal pigment epithelium; SW‐FAF, short‐wavelength fundus autofluorescence

## 5 CONCLUSIONS AND FUTURE DIRECTIONS

Our work provides a comprehensive overview of the sequence variant spectrum of *CNGB1‐*linked RP in the largest cohort reported to date, including 22 novel identified variants and sets the basis for the recruitment of patients in future therapeutic trials of gene augmentation or cell therapies. Our study also provides a complete phenotypic characterization of a relatively large cohort of subjects affected by this form of RP. However, natural history studies with longitudinal follow‐up are warranted to better define prognostic factors and window of intervention.

## WEB RESOURCES

RETNET: https://sph.uth.edu/retnet/

HGMD® Professional 2017.4: http://www.hgmd.cf.ac.uk/ac/index.php

Leiden Open Variation Database (LOVD V.3.0): https://www.lovd.nl/

ClinVar: https://www.ncbi.nlm.nih.gov/clinvar/

Mutation Taster: http://www.mutationtaster.org/

Sorting Intolerant From Tolerant SIFT: https://sift.bii.a-star.edu.sg/

Polymorphism Phenotyping v2 Polyphen‐2: http://genetics.bwh.harvard.edu/pph2/

Splice Site Prediction by Neural Network, NNSPLICE: https://www.fruitfly.org/seq_tools/splice.html

Human Splicing Finder v.2.4.1, HSF: http://www.umd.be/HSF/

HomoloGene: https://www.ncbi.nlm.nih.gov/homologene

UCSC Genome Browser: https://genome.ucsc.edu/index.html).

ANNOVAR: http://annovar.openbioinformatics.org/en/latest/

Genome Aggregation Database (gnomAD): https://gnomad.broadinstitute.org/

UniProt Align tool: https://www.uniprot.org/align/.

## Supporting information

Supporting information.

## Data Availability

The data that support the findings of this study will be openly available in LOVD at https://www.lovd.nl/ after the acceptance of the study.
