# Supplementary material for: CNGB1‐related rod‐cone dystrophy: A mutation review and update
Source: Hum Mutat. 2021 May 16;42(6):641–66. doi: 10.1002/humu.24205 (PMC8218941; doi:10.1002/humu.24205)
Supplement: Supplementary file 1 — Supporting information. [file HUMU-42-641-s001.pdf]

CNGB1-related rod-cone dystrophy: a mutation review and update.

Nassisi et al.

Supplemental Material

Contents:

Table S1. *In silico* analysis performed on the *CNGB1* variants included in the study.

Table S2. *CNGB1* conservation analysis (without GARP domain) on homologous genes

Table S3. Conservation analysis of the novel missense variants on *CNGB1,* described in the study.

Table S4. Conservation analysis of the novel canonical and non-canonical splice site variants on *CNGB1,* described in the study.

Table S5. Additional clinical phenotype data of the subjects carrying likely disease-causing *CNGB1* variants included in the study**.**

References

Table S1. *In silico* analysis performed on the *CNGB1* variants included in the study. PhyloP range: -14.1;6.4. Grantham score range: 0;215. CNBD: cyclic nucleotide binding domain; CaMBD: Calmodulin binding domain

Genomic start position (hg19)	Exon/ Intron	cDNA (NM_001297.5)	Protein change (NP_001288.3)	Protein domain involved/putative functional consequence	rs#; gnomAD: frequency (allele count/total alleles/number of homozygous)	PhyloP	Grantham	Pathogenic prediction for missense changes			Effect on splicing prediction		
								SIFT (score)	MutuationTaster (prob)	PolyPhen 2 (score)	MaxEnt	NNSplice	SSF
58001027	IVS2	c.159+5G>A	p.?	Possible effect on splicing leading to a shorter/longer protein product or NMD	no rs#; gnomAD: absent	-	-	-	-	-	-92.20%	-95.70%	-14.90%
57998386	IVS3	c.217+5G>C	p.?	Possible effect on splicing leading to a shorter/longer protein product or NMD	rs527236060; gnomAD: 0.0046% (13/280826/0)	-	-	-	-	-	-70.10%	-51.70%	-14.70%
57998062	4	c.262C>T	p.(Gln88*)	Premature stop codon leading to a shorter protein product with only the N-terminus fragment or NMD	rs878853394; gnomAD: absent	-	-	-	Disease Causing (1)	-	-	-	-
57996944	5	c.315G>A	p.(Trp105*)	Premature stop codon leading to a shorter protein product with only the N-terminus fragment or NMD	rs1420628245; gnomAD: 0,0004% (1/248694/0)	-	-	-	Disease Causing (1)	-	-	-	-
57996913	5	c.346C>T	p.(Gln116*)	Premature stop codon leading to a shorter protein product with only the N-terminus fragment or NMD	rs367677784; gnomAD: 0.0004% (1/249010/0)	-	-	-	Disease Causing (1)	-	-	-	-

57996773	6	c.385del	p.(Leu129 Trpfs*148)	Premature stop codon leading to a shorter protein product with only the N-terminus fragment or NMD	no rs#; gnomAD: absent	-	-	-	Disease Causing (1)	-	-	-	-
57996515	IVS6	c.413-1G>A	p.[= ; Cys139Ala fs*138]#	Creation of a cryptic splice site downstream, leading to the loss of the first nucleotide of exon 7, hence to a frameshift	rs189234741; gnomAD: 0.0075% (21/280578/0)	-	-	-	-	-	-100.00%	-100.00%	-100.00%
57994756	8	c.522dup	p.(Lys175 Glnfs*4)	Premature stop codon leading to a shorter protein product with only the N-terminus fragment or NMD	no rs#; gnomAD: 0.0004% (1/248226/0)	-	-	-	Disease Causing (1)	-	-	-	-
57994743	IVS8	c.534+1G>A	p.?	Possible effect on splicing leading to a shorter/longer protein product or NMD	rs200862689; gnomAD: 0.0036% (9/248004/0)	-	-	-	-	-	-100.00%	-100.00%	-100.00%
57993889	10	c.664C>T	p.(Gln222 *)	Premature stop codon leading to a shorter protein product with only the N-terminus fragment or NMD	rs570828500; gnomAD: 0.0005% (1/210288/0)	-	-	-	Disease Causing (1)	-	-	-	-
57992344	11	c.807G>C	p.(Gln269 His)	GARP Domain	no rs#; gnomAD: absent	0,69	24	Deleteri ous (0)	Polymorp hism (0.997)	Probably Damaging (0.995)	-	-	-
57992319	11	c.832G>T	p.(Glu278 *)	Premature stop codon leading to a shorter protein product with only the N-terminus fragment or NMD	no rs#; gnomAD: absent	-	-	-	Disease Causing (1)	-	-	-	-
57993790	IVS10	c.761+2T>A	p.?	Possible effect on splicing leading to a	no rs#; gnomAD: absent	-	-	-	-	-	-100.00%	-100.00%	-100.00%

				shorter/longer protein product or NMD									
57984446	IVS12	c.875-2A>C	p.?	Possible effect on splicing leading to a shorter/longer protein product or NMD	no rs#; gnomAD: absent	-	-	-	-	-	-100.00%	-100.00%	-100.00%
57984428	IVS12	c.875-5_891del	p.?	Premature stop codon leading to a shorter protein product with only the N-terminus fragment or NMD	no rs#; gnomAD: absent	-	-	-	-	-	-100.00%	-100.00%	-100.00%
57984380	13	c.939G>A	p.(Trp313 *)	Premature stop codon leading to a shorter protein product with only the N-terminus fragment or NMD	no rs#; gnomAD: absent	-	-	-	Disease Causing (1)	-	-	-	-
57984367	13	c.952C>T	p.(Gln318 *)	Premature stop codon leading to a shorter protein product with only the N-terminus fragment or NMD	rs372504780; gnomAD: 0.0008% (2/249588/0)	-	-	-	Disease Causing (1)	-	-	-	-
57974234	IVS14	c.1122-9G>A	p.[=; Glu374Glu fs*7]‡	Causes retention of 7 bp of intron 14 resulting in a frameshift with stop codon 7AA downstream of exon 15.	rs750225817; gnomAD: 0.0024% (5/208958/0)	-	-	-	-	-	-100.00%	-10.90%	-100.00%
57974160	15	c.1187G>A	p.(Arg396 Gln)	Linker GARP-Channel domain	rs370327030; gnomAD: 0.0046% (10/216252/0)	-0,68	43	Tolerated (0.58)	Polymorphism (1)	Benign (0)	-	-	-
57973487	16	c.1219dup	p.(Glu407 Glyfs*12)	Premature stop codon leading to a shorter protein product with only the N-terminus fragment or NMD	no rs#; gnomAD: absent	-	-	-	Disease Causing (1)	-	-	-	-

57973394	16	c.1312C>T	p.(Gln438 *)	Premature stop codon leading to a shorter protein product with only the N-terminus fragment or NMD	rs776632878; gnomAD: 0.0008% (2/246622/0)	-	-	-	Disease Causing (1)	-	-	-	-
57973387	16	c.1319G>A	p.(Trp440 *)	Premature stop codon leading to a shorter protein product with only the N-terminus fragment or NMD	no rs#; gnomAD: absent	-	-	-	Disease Causing (1)	-	-	-	-
57965773	17	c.1382C>T	p.(Thr461 Met)	Linker GARP-Channel domain	rs147593839; gnomAD: 0.8955% (2513/280628/44)	3,35	81	Tolerate d (0.13)	Polymorp hism (0.888)	Probably Damaging (1)	-	-	-
57957231	18	c.1589C>G	p.(Pro530 Arg)	Linker GARP-Channel domain	rs201553871; gnomAD: 0.0401% (117/280670/2)	0,85	103	Tolerate d (0.17)	Disease causing (0.073)	Probably Damaging (0.998)	-	-	-
57957207	18	c.1613G>A	p.(Trp538 *)	Premature stop codon leading to a shorter protein product with only the N-terminus fragment or NMD	rs1258900049; gnomAD: 0.0004% (1/249196/0)	-	-	-	Disease Causing (1)	-	-	-	-
57954451	IVS18	c.1644-3C>G	p.?	Possible effect on splicing leading to a shorter/longer protein product or NMD	no rs#; gnomAD: 0.0004% (1/247932/0)	-	-	-	-	-	-100.00%	-99.90%	-13.70%
57954363	19	c.1729del	p.(Glu577 Serfs*6)	Premature stop codon leading to a shorter protein product with only the N-terminus fragment or NMD	no rs#; gnomAD: absent	-	-	-	Disease Causing (1)	-	-	-	-
57954286	IVS19	c.1801+5G>A	p.?	Possible effect on splicing leading to a shorter/longer protein product or NMD	rs375550800; gnomAD: 0.0025% (7/280852/0)	-	-	-	-	-	-100.00%	-98.50%	-16.40%

57953064	20	c.1896C>A	p.(Cys632 *)	Premature stop codon leading to a shorter protein product with only the N-terminus fragment or NMD	rs774264204; gnomAD: 0.0004% (1/249562/0)	-	-	-	Disease Causing (1)	-	-	-	-
57953043	20	c.1917G>A	p.(Trp639 *)	Premature stop codon leading to a shorter protein product with only the N-terminus fragment or NMD	rs183443649; gnomAD: 0.0004% (1/249570/0)	-	-	-	Disease Causing (1)	-	-	-	-
57953019	20	c.1941C>A	p.(Ser647 Arg)	Linker GARP-Channel domain	rs1187092335; gnomAD: 0.0008% (2/249554/0)	-	-	Deleteri ous (0.01)	Disease Causing (1)	Probably Damaging (0,997)	-	-	-
57953001	IVS20	c.1957+2T>G	p.?	Possible effect on splicing leading to a shorter/longer protein product or NMD	rs755398007; gnomAD: 0.0012% (3/249502/0)	-	-	-	-	-	-100.00%	-100.00%	-100.00%
57951234	21	c.2093_2104du p	p.(Cys698 _Ile701du p)	Longer protein product with possible effect on the overall structure of the protein	No rs#; gnomAD: absent	-	-	-	Disease Causing (1)	-	-	-	-
57951242	21	c.2096A>G	p.(Asp699 Gly)	TD2	rs878853393; gnomAD: absent	3,27	94	Deleteri ous (0)	Disease Causing (1)	Probably Damaging (1)	-	-	-
57951166	IVS21	c.2166+6T>C	p.?	Possible effect on splicing leading to a shorter/longer protein product or NMD	rs771401261; gnomAD: 0.0032% (9/274684/0)	-	-	-	-	-	-48.6%	-62.7%	-7.8%
57950065	22	c.2185C>T	p.(Arg729 *)	Premature stop codon leading to a shorter protein product with only the N-terminus fragment or NMD	no rs#; gnomAD: 0.0011% (3/280968/0)	-	-	-	Disease Causing (1)	-	-	-	-

57950040	22	c.2210G>A	p.(Arg737 His)	TD3	rs764107600; gnomAD: 0.0018% (5/280948/0)	4,81	29	Deleteri ous (0.03)	Disease Causing (1)	Probably Damaging (0.999)	-	-	-
57949241	IVS22	c.2218-2A>G	p.?	Possible effect on splicing leading to a shorter/longer protein product or NMD	no rs#; gnomAD: absent	-	-	-	-	-	-100.00%	0.00%	-100.00%
57949199	23	c.2258T>A	p.(Leu753 *)	Premature stop codon leading to a shorter protein product with only the N-terminus fragment or NMD	no rs#; gnomAD: absent	-	-	-	Disease Causing (1)	-	-	-	-
57949173	23	c.2284C>T	p.(Arg762 Cys)	Linker TD3-TD4	rs1028371920; gnomAD: 0.0007% (2/280610/0)	1,58	180	Deleteri ous (0)	Disease Causing (1)	Probably Damaging (1)	-	-	-
57949172	23	c.2285G>A	p.(Arg762 His)	Linker TD3-TD4	rs760373259; gnomAD: 0.0016% (4/249018/0)	5,29	29	Deleteri ous (0)	Disease Causing (1)	Probably Damaging (1)	-	-	-
57949164	23	c.2293C>T	p.(Arg765 Cys)	Linker TD3-TD4	rs771833874; gnomAD: 0.002% (5/249044/0)	5,29	180	Deleteri ous (0)	Disease Causing (1)	Probably Damaging (1)	-	-	-
57949163	23	c.2294G>T	p.(Arg765 Leu)	Linker TD3-TD4	no rs#; gnomAD: absent	5,29	102	Deleteri ous (0)	Disease Causing (1)	Probably Damaging (1)	-	-	-
57946900	IVS23	c.2305-2A>G	p.?	Possible effect on splicing leading to a shorter/longer protein product or NMD	no rs#; gnomAD: 0.0004% (1/249138/0)	-	-	-	-	-	-100.00%	-100.00%	-100.00%
57912979	24	c.2320G>A	p.(Glu774 Lys)	Linker TD3-TD4	rs1286857064; gnomAD: 0.0004% (1/249434/0)	7,64	56	Tolerate d (0.09)	Disease Causing (1)	Possibly Damaging (0.810)	-	-	-
57946850	24	c.2353A>T	p.(Lys785 *)	Premature stop codon leading to a shorter protein product with only the N-terminus fragment or NMD	no rs#; gnomAD: absent	-	-	-	Disease Causing (1)	-	-	-	-

57946842	24	c.2361C>A	p.(Tyr787*)	Premature stop codon leading to a shorter protein product with only the N-terminus fragment or NMD	rs367678786; gnomAD: 0.0012% (3/249544/0)	-	-	-	Disease Causing (1)	-	-	-	-
57945656	IVS25	c.2492+1G>A	p.?	Possible effect on splicing leading to a shorter/longer protein product or NMD	rs530551814; gnomAD: absent	-	-	-	-	-	-100.00%	-100.00%	-100.00%
57911751	IVS25	c.2492+2T>G	p. ?	Possible effect on splicing leading to a shorter/longer protein product or NMD	no rs#; gnomAD: absent	-	-	-	-	-	-100.00%	-100.00%	-100.00%
57938777	IVS25	c.2493-2_2495delinsGGC	p.?	Possible effect on splicing leading to a shorter/longer protein product or NMD	no rs#; gnomAD: absent	-	-	-	Disease Causing (1)	-	-100.00%	-100.00%	-100.00%
57938781	IVS25	c.2493-2A>G	p.?	Possible effect on splicing leading to a shorter/longer protein product or NMD	rs745319716; gnomAD: 0.002% (5/249394/0)	-	-	-	-	-	-100.00%	-100.00%	-100.00%
57938771	26	c.2501G>T	p.(Arg834Leu)	Linker TD4-TD5	rs761295014; gnomAD: 0,0014% (4/280872/0)	5,69	102	Deleterious (0)	Disease Causing (1)	Possibly Damaging (0.801)	-	-	-
57938764	26	c.2508C>A	p.(Tyr836*)	Premature stop codon leading to a shorter protein product with only the N-terminus fragment or NMD	no rs#; gnomAD: 0.0011% (3/280910/0)	-	-	-	Disease Causing (1)	-	-	-	-
57938748	26	c.2524dup	p.(Thr842Asnfs*10)	Premature stop codon leading to a shorter protein product with only the N-terminus fragment or NMD	rs527236061; gnomAD: absent	-	-	-	-	-	-	-	-

57938732	26	c.2540G>A	p.(Gly847 Glu)	Linker Pore-TD6	no rs#; gnomAD: 0.0008% (2/249580/0)	5,69	98	Deleteri ous (0)	Disease Causing (1)	Probably Damaging (1)	-	-	-
57938730	26	c.2542_2543ins A	p.(Gly848 Glufs*4)	Linker Pore-TD6	no rs#; gnomAD: absent	-	-	-	Disease Causing (1)	-	-	-	-
57938728	26	c.2544dup	p.(Leu849 Alafs*3)	Premature stop codon leading to a shorter protein product with only the N-terminus fragment or NMD	rs760430056; gnomAD: (0.0036% 10/280958/0)	-	-	-	Disease Causing (1)	-	-	-	-
57938727	26	c.2545dup	p.(Leu849 Profs*3)	Premature stop codon leading to a shorter protein product with only the N-terminus fragment or NMD	no rs#; gnomAD: absent	-	-	-	Disease Causing (1)	-	-	-	-
57938717	26	c.2555C>T	p.(Pro852 Leu)	TD6	rs768202610; gnomAD: 0,0014% (4/280960/0)	5,69	98	Deleteri ous (0)	Disease Causing (1)	Probably Damaging (1)	-	-	-
57938697	26	c.2575G>A	p.(Val859I le)	TD6	rs1308416277; gnomAD 0,0007% (2/280982/0)	0,85	29	Tolerate d (0.33)	Disease Causing (0.989)	Benign (0.057)	-	-	-
57938669	26	c.2603G>A	p.(Gly868 Asp)	TD6	rs770961534; gnomAD: 0,0008% (2/249582/0)	4,16	94	Deleteri ous (0)	Disease Causing (1)	Probably Damaging (1)	-	-	-
57937858	26	c.2662G>A	p.(Ala888 Thr)	TD6	rs368328328; gnomAD: 0,022% (63/280510/0)	2,87	58	Tolerate d (0.17)	Disease Causing (1)	Possibly Damaging (0.722)	-	-	-
57937844	27	c.2676C>A	p.(Tyr892 *)	Premature stop codon leading to a shorter protein product with only the N-terminus fragment or NMD	no rs#; gnomAD: absent	-	-	-	Disease Causing (1)	-	-	-	-
57937839	27	c.2681G>A	p.(Arg894 His)	C-linker	rs146762538; gnomAD: 0.0442% (124/280786/0)	5,53	29	Deleteri ous (0.01)	Disease Causing (1)	Possibly Damaging (0.953)	-	-	-
57937760	27	c.2760G>A	p.(Trp920 *)	Premature stop codon leading to a shorter protein	rs776312649; gnomAD: 0.0008% (2/249494/0)	-	-	-	Disease Causing (1)	-	-	-	-

				product with only the N-terminus fragment or NMD									
57937755	27	c.2762_2765del	p.(Tyr921 Cysfs*15)	Premature stop codon leading to a shorter protein product with only the N-terminus fragment or NMD	no rs#; gnomAD: absent	-	-	-	Disease Causing (1)	-	-	-	-
57937757	27	c.2763C>G	p.(Tyr921 *)	Premature stop codon leading to a shorter protein product with only the N-terminus fragment or NMD	no rs#; gnomAD: absent	-	-	-	Disease Causing (1)	-	-	-	-
57935256	28-29	c.2777-?_2958+?del	p.?	Premature stop codon leading to a shorter protein product with only the N-terminus fragment or NMD	no rs#; gnomAD: absent	-	-	-	Disease Causing (1)		0.00%	0.00%	0.00%
57937725	IVS27	c.2794+1G>A	p.?	Possible effect on splicing leading to a shorter/longer protein product or NMD	rs770011113; gnomAD: 0.0092% (23/249286/0)	-	-	-	-	-	-100.00%	-100.00%	-100.00%
57935519	28	c.2805del	p.(Glu935 Aspfs*2)	Premature stop codon leading to a shorter protein product with only the N-terminus fragment or NMD	no rs#; gnomAD: 0.0008% (2/249354/0)	-	-	-	Disease Causing (1)	-	-	-	-
57935457	28	c.2867del	p.(Ile956T hrfs*15)	Premature stop codon leading to a shorter protein product with only the N-terminus fragment or NMD	rs1231250334; gnomAD: 0.0008% (2/249570/0)	-	-	-	Disease Causing (1)	-	-	-	-
57935435	28	c.2888_2889del	p.(Phe963 Serfs*4)	Premature stop codon leading to a shorter protein product with only	no rs#; gnomAD: absent	-	-	-	Disease Causing (1)	-	-	-	-

				the N-terminus fragment or NMD									
57935346	IVS28	c.2893-7G>A	p.?	Possible effect on splicing leading to a shorter/longer protein product or NMD	rs749199721; gnomAD: 0.005% (14/280976/0)	-	-	-	-	-	-54.70%	0.50%	-100%
57935339	29	c.2893G>A	p.(Gly965 Ser)	C-linker; Possible effect on splicing leading to a shorter/longer protein product or NMD	rs559591083; gnomAD: absent	4,08	56	Tolerate d (0.08)	Disease Causing (1)	Possibly Damaging (0.866)	-15.70%	-77.50%	-5.40%
57935311	29	c.2921T>G	p.(Met974 Arg)	CNBD	Rs752967885; gnomAD: 0.0035% (10/280974/0)	3,19	91	Deleteri ous (0)	Disease Causing (1)	Probably Damaging (0.988)	-	-	-
57935275	29	c.2957A>T	p.(Asn986 Ile)	CNBD	rs201162411; gnomAD: 0.1182% (332/280924/0)	3,27	149	Deleteri ous (0.01)	Disease Causing (1)	Probably Damaging (0.983)	-	-	-
57935266	29	c.2966T>A	p.(Val989 Glu)	CNBD	rs201162411; gnomAD: 0.0014% (4/280866/0)	4,73	121	Deleteri ous (0)	Disease Causing (1)	Possibly Damaging (0.998)	-	-	-
57931817	30	c.2978G>T	p.(Gly993 Val)	CNBD	rs121918532; gnomAD: absent	5,94	109	Deleteri ous (0.01)	Disease Causing (1)	Probably Damaging (1)	13.10%	0.30%	0.00%
57931815	30	c.2980G>T	p.(Glu994 *)	Premature stop codon leading to a shorter protein product with only the N-terminus fragment or NMD	no rs#; gnomAD: absent	-	-	-	Disease Causing (1)	-	0.00%	-0.20%	0.00%
57931745	30	c.3044_3050del	p.(Gly101 5Valfs*4)	Premature stop codon leading to a shorter protein product with only the N-terminus fragment or NMD	no rs#; gnomAD: absent	-	-	-	Disease Causing (1)	-	-	-	-
57931394	30	c.3131_3149del	p.(Ala104 4Glyfs*13)	Premature stop codon leading to a shorter protein product with only	rs1365926616; gnomAD: 0.0014% (4/280916/0)	-	-	-	Disease Causing (1)	-	-	-	-

				the N-terminus fragment or NMD									
57931401	31	c.3139_3142dup	p.(Ala1048Glyfs*13)	Premature stop codon leading to a shorter protein product with only the N-terminus fragment or NMD	rs756806434; gnomAD: 0.0036% (10/280876/0)	-	-	-	Disease Causing (1)	-	-	-	-
57931393	31	c.3150del	p.(Phe1051Leufs*12)	Premature stop codon leading to a shorter protein product with only the N-terminus fragment or NMD	rs753353134; gnomAD: 0.0014% (4/280902/0)	-	-	-	Disease Causing (1)	-	-	-	-
57921758	IVS32	c.3462+1G>A	p.[=;Arg1081Argfs*68]†	Possible effect on splicing leading to a shorter/longer protein product or NMD	no rs#; gnomAD: absent	-	-	-	-	-	-100.00%	-100.00%	-100.00%

#: Effect validated by means of minigene assay in (Saqib et al., 2015). †: Effect validated by means of minigene assay by (Becirovic et al., 2010). ‡: Effect validated by means of *in vitro* assay by (Petersen-Jones et al., 2018)

Table S2. *CNGB1* conservation analysis (without GARP domain) on homologous genes performed using the Alignment tool on UniProt (available at: https://www.uniprot.org/align/). The novel missense variants found in the study are highlighted in red.

```
Q14028|CNGB1_H.SAPIENS       EEEEEEEEEEEVTEVLLDSCVVSQVGVGQSEEDGTRPQSTSDQKLWEEVGEEAKKEAEEK 60
Q9NQW8|CNGB3_H.SAPIENS       ---------------MFKSLT-KVNKVKPIGENNENEQSSR------------------- 25
Q9JJZ9|CNGB3_M.MUSCULUS      ---------------MLKSLTVKFNKVNPMEGRMEKK----------------------- 22
Q8MJD7|CNGB3_C.LUPUS         ---------------MFKSLTIKSNKVKPREENDEN------------------------ 21
W5PPU7|CNGB3_O.ARIES         ------------------------------------------------------------
A0A1D5PGA2|CNGB3_G.GALLUS    ------------------------------------------------------------
A0A2R8RTW7|cngb3.2_D.RERIO   ------------------------------------------------------------
A0A0R4IL01|cngb3.1_D.RERIO   ------------------------------------------------------------
G5EEE2|tax-2_C.ELEGANS       --------------------------------------------MYQ-VPKRAK------ 9

Q14028|CNGB1_H.SAPIENS       AKEEAEEVAEEEAEKEPQDWAETKE--EPEAEAEAASSGVPATKQHP-EVQVEDTDADSC 117
Q9NQW8|CNGB3_H.SAPIENS       ---------------------RNEE--GSHPS----NQSQQTTAQEENKGEEKSLKTKST 58
Q9JJZ9|CNGB3_M.MUSCULUS      ------------------------L--CPNLS----SLSQPTIAQGDNQSEKEPLR-SRT 51
Q8MJD7|CNGB3_C.LUPUS         -----------------------KQ--DPDPS----NQPQQSTRQGENKSENKSLQTKMT 52
W5PPU7|CNGB3_O.ARIES         ------------------------------------------------------------
A0A1D5PGA2|CNGB3_G.GALLUS    ---------------------------------------------------------AHC 3
A0A2R8RTW7|cngb3.2_D.RERIO   ----------------VSSFIRKILAAAPEPAAP-AKEPEPAAAKEPEQA---------A 34
A0A0R4IL01|cngb3.1_D.RERIO   -------------------------------------------------P---------A 2
G5EEE2|tax-2_C.ELEGANS       -----TNLARE-IRKREFSYVDRQKA----------SKPTQLSEKEWKSP---------- 43

Q14028|CNGB1_H.SAPIENS       PLMAEENPPSTVLPPPSPAKSDTLIVPSSASGTHRKK--LPSEDD-EAEELKALSPAESP 174
Q9NQW8|CNGB3_H.SAPIENS       PVTSEEPHTN---------IQDKLSKK--NS--SGDLTTNPDPQN-AAEPTGTVPEQKEM 104
Q9JJZ9|CNGB3_M.MUSCULUS      PITFEKSHSK---------E-DNSTGE--NS--LRDFTPNPDPEC-RAELTRTMAEMEKT 96
Q8MJD7|CNGB3_C.LUPUS         PVTFEESHAK---------MQDKISEK--NS--LRDLTTNPNHQH-PTESKGAMSEQKEM 98
W5PPU7|CNGB3_O.ARIES         ------------------------------------------------------------
A0A1D5PGA2|CNGB3_G.GALLUS    WLTFSCTSTN---------TSRSISSA--QSSSHSSLQRSPNAWQ------SWLA----L 42
A0A2R8RTW7|cngb3.2_D.RERIO   P--AKEPE------PAAPAKEPEPA--------------APAPAK-EPEP-AAPAPAKEP 70
A0A0R4IL01|cngb3.1_D.RERIO   P--GSAPA------PA-----P-----------------APAPGS-APAP----APASAP 27
G5EEE2|tax-2_C.ELEGANS       ----RSEDSFDLLDPANASKEPSAS--------TR---PLPYPPTRPPEVVIQIDEVESP 88
```

```
Q14028|CNGB1_H.SAPIENS      V-VAW-----------SDPTTPKDTDGQDR-------AASTASTNSAIINDRLQELVKLF 216
Q9NQW8|CNGB3_H.SAPIENS      DPGK------------EGPNSP------QN-------KPPAAPVINEYADAQLHNLVKRM 139
Q9JJZ9|CNGB3_M.MUSCULUS     RTGK------------ERPVSF------KT-------KVLETSIINEYTDAHLHNLVERM 131
Q8MJD7|CNGB3_C.LUPUS        ETGK------------EGLVSP------KS-------KPLGVPVINEYADAQLHNLVRRM 133
W5PPU7|CNGB3_O.ARIES        -----------------------------H-------PKSKSPVINEYADAQLHSLVRRL 24
A0A1D5PGA2|CNGB3_G.GALLUS   QNGLC-----------LHSNNT------QN-------ATSQGFQTNEYADAQLQEIVRKM 78
A0A2R8RTW7|cngb3.2_D.RERIO  ---EP-----------AAPAPAAKPEGTPA-------DPPRPILISEEIDTHLIEMIQKL 109
A0A0R4IL01|cngb3.2_D.RERIO  APAPA-----------SAPAPAPAEDGPPS-------PPTPPVVYYRYTDDQLRDIVKKM 69
G5EEE2|tax-2_C.ELEGANS      ILGLIDETDDHELDGRLDPASSFDANSLSATRASSIIEDDVRSQISFIMRERLHSIAKEV 148


Q14028|CNGB1_H.SAPIENS      KERTEKVKEKLIDPDV-TSDEESPKPSPAKKAPEP---------------------A--- 250
Q9NQW8|CNGB3_H.SAPIENS      RQRTALYKKKLVEGD-LSSPEASPQTAKPTAV---------------------------- 170
Q9JJZ9|CNGB3_M.MUSCULUS     RERTALYKKTLTEEENFPEVEASSQTAMSTNI---------------------------- 163
Q8MJD7|CNGB3_C.LUPUS        RQRTMLYKKKLAEGD-ISSPEASPQTAKPTAV---------------------------- 164
W5PPU7|CNGB3_O.ARIES        RQRTDFYKRKLVEAD-MSSPESSPQTAKPTAV---------------------------- 55
A0A1D5PGA2|CNGB3_G.GALLUS   RERATAYKEKLKDPV-LSSPEGSPTPPPPPKEEKK---------------------E--- 113
A0A2R8RTW7|cngb3.2_D.RERIO  RQRTEQFKEKVIDPY-ASSPERSPPVTPVLRKDDYYKKRQEEEERIRKEEE-KKRKEEEA 167
A0A0R4IL01|cngb3.2_D.RERIO  RERTELYKDKIVDPY-ASSPERTPPVTPVYRKEDWIRKQEEE--RIKREEAEQKKKEDDA 126
G5EEE2|tax-2_C.ELEGANS      HRRTSAVREDLIRET----PEDTVSM--ASNV---------------------------- 174


Q14028|CNGB1_H.SAPIENS      --------PDTKPAEAEPVE------------------EEHY--CDMLCCKFKHRPWKKY 282
Q9NQW8|CNGB3_H.SAPIENS      --------P-PVKESDDK-P------------------TEHY--YRLLWFKVKKMPLTEY 200
Q9JJZ9|CNGB3_M.MUSCULUS     --------S-PKQENNSKLK------------------EHQ----DTFSFKPQRVPVKEH 192
Q8MJD7|CNGB3_C.LUPUS        --------P-STQESNAKLK------------------EEHY--YHILCFKFQKMPLTEY 195
W5PPU7|CNGB3_O.ARIES        --------S-STQESSAKLK------------------EEHS----------QKAPLIEY 78
A0A1D5PGA2|CNGB3_G.GALLUS   --------E-KKEEAEAKPE------------------EDHY--CDMLCCKFKKPPLKKY 144
A0A2R8RTW7|cngb3.2_D.RERIO  KKKKEEAEKKKKEEEEKKKKEEEEK--KKEQAGKPK--KSIFSKVNFTCVDFVLKPFEMR 223
A0A0R4IL01|cngb3.2_D.RERIO  KRAAA---KKEKEEKEKKEKEEKLKAEKEAADKEAA--EAMFPKIKCTCIDTLLKPFEDK 181
G5EEE2|tax-2_C.ELEGANS      ----------PKQNEHRP-S-------LMSLIGLQNRSESPTVDTVKNCFGF-------- 208
```

```
                                   p.(S647R)
Q14028|CNGB1_H.SAPIENS       ----QFPQSIDPLTNLMYVLWLFFVVMAWNWNCWLIPVRWAFPYQTPDNIHHWLLMDYLC 338
Q9NQW8|CNGB3_H.SAPIENS       LKRIKLPNSIDSYTDRLYLLWLLLVTLAYNWNCCFIPLRLVFPYQTADNIHYWLIADIIC 260
Q9JJZ9|CNGB3_M.MUSCULUS      LRRMILPRSIDSYTDRVYLLWLLLVTIAYNWNCWLLPVRLVFPCQTPDNKNYWIITDIVC 252
Q8MJD7|CNGB3_C.LUPUS         LKRFRLPGSIDSYTDRLYLLWLLLVTIAYNWNCWLIPLRLVFPYQTPDNTHYWFITDITC 255
W5PPU7|CNGB3_O.ARIES         LKRLRLPKSIDSYTDRFYLLWLLLVTIAFNWNCWFIPLRMVFPYQTPHNTHYWLITDLVC 138
A0A1D5PGA2|CNGB3_G.GALLUS    MTYLKLPDSIDSYTDRRYVAWLMLVTVAYNWNCWFIPLRFVFPYQTPSNTIYWFAIDIIC 204
A0A2R8RTW7|cngb3.2_D.RERIO   M-DNRIGKSIDPFTDRRYITWLSMVTIAFNYNLWFVPVRMAFPYHSPEVVPLWFTLDIIA 282
A0A0R4IL01|cngb3.2_D.RERIO   M-DSYLGATIDPFTDRRYIKWLSVVTVAFNYNVWLATARLCFPYHTPATIPFWILFDFLA 240
G5EEE2|tax-2_C.ELEGANS       ----SLKGTFHPY-GRFYMTWLSLVTLCFLFNAFCIPLRSSYPYQTADNWMYWFIVDYSC 263

Q14028|CNGB1_H.SAPIENS       DLIYFLDITVFQTRLQFVRGGDIITDKKDMRNNYLKSRRFKMDLLSLLPLDFLYLKVGVN 398
Q9NQW8|CNGB3_H.SAPIENS       DIIYLYDMLFIQPRLQFVRGGDIIVDSNELRKHYRTSTKFQLDVASIIPFDICYLFFGFN 320
Q9JJZ9|CNGB3_M.MUSCULUS      DIIYLCDILLIQPRLQFVRGGEIIVDSNELKRNYRSSTKFRMDVASLLPFEVLYIFFGVN 312
Q8MJD7|CNGB3_C.LUPUS         DIIYLCDMLLIQPRLQFIKGGDIMVDSNELKRHYRSSTKFQLDVASVMPFDVFYLFFGFN 315
W5PPU7|CNGB3_O.ARIES         DTIYLFDLLLIQPRLQFMRGGDIIVDSNELKRHYRNSTKFQLDMASIMPFDVFYLFFGFN 198
A0A1D5PGA2|CNGB3_G.GALLUS    DICYLCDLLVFQPRVQFLRGGDIISDKVEMKKFYHSTIKFRLDLISVLPFDVLYFFFGFN 264
A0A2R8RTW7|cngb3.2_D.RERIO   DFIYIFDMIFFQPRLQFSKGGDIIYDREVIKKKYRESGRFQNDVIAVLPIDLLYIPFGFN 342
A0A0R4IL01|cngb3.2_D.RERIO   DLVNVIDITMFQPRLQFVKAGDIIKDRVQTKQNYRESARFQTDLISIIPFDLLCFQFGFT 300
G5EEE2|tax-2_C.ELEGANS       DLVYVIDMLLIKPRLRFTRGGIQVKIYKDTQRHYLMTRTFKLDILSILPTDLMYFFFGKM 323

                                          p.(E774K)
Q14028|CNGB1_H.SAPIENS       PLLRLPRCLKYMAFFEFNSRLESILSKAYVYRVIRTTAYLLYSLHLNSCLYYWASAYQGL 458
Q9NQW8|CNGB3_H.SAPIENS       PMFRANRMLKYTSFFEFNHHLESIMDKAYIYRVIRTTGYLLFILHINACVYYWASNYEGI 380
Q9JJZ9|CNGB3_M.MUSCULUS      PIFRANRILKYTSFFEFNHHLESIMDKAYVYRVIRTTGYLLFLLHINACVYYWASDYEGI 372
Q8MJD7|CNGB3_C.LUPUS         PVFRMNRILKYTSFFEFNHHLESIMDKAYIYRVIRTTGYLLYTLHINACIYYWASDYEGI 375
W5PPU7|CNGB3_O.ARIES         PIFRTNRMLKYTSFFEFNHHLESIMNKAYIYRVIRTTGYLLFTLHINACMYYWASSYEGI 258
A0A1D5PGA2|CNGB3_G.GALLUS    PAFRVNRMLKHNTFFEFNDRLEAILDKAYIYRVIRTTGYLLFILHINACLYYWASDYEGL 324
A0A2R8RTW7|cngb3.2_D.RERIO   SVFRLNRLMKVESFFEFSDRLEGLLTRAYIWRVIRTIGYLLFILHLNACLYYVASVSQGI 402
A0A0R4IL01|cngb3.2_D.RERIO   SFFRLNRFMRYQSFFEFSDRLESIMAKAYIWRVGRTTGYLLYCLHINSCLYYVASEYEGL 360
G5EEE2|tax-2_C.ELEGANS       PIWRINRVLKINSFWLLFDMLDNSFANPYAIRIARTLSYMIYIIHCNSCVYYKLSALQAF 383
```

```
                                                   p.(R834L)          p.(P852L) p.(V859I)
Q14028|CNGB1_H.SAPIENS      GS-----------THWVYDGVGNS-YIRCYYFAVKTLITIGGLPDPKTLFEIVFQLLNYF 506
Q9NQW8|CNGB3_H.SAPIENS      GT-----------TRWVYDGEGNE-YLRCYYWAVRTLITIGGLPEPQTLFEIVFQLLNFF 428
Q9JJZ9|CNGB3_M.MUSCULUS     GS-----------TKWVYNGEGNK-YLRCFYWAVRTLITIGGLPEPQTSFEIVFQFLNFF 420
Q8MJD7|CNGB3_C.LUPUS        GS-----------TKWVYNGEGNK-YLRCYYWAVRTLITIGGLPEPQTSFEIVFQLLNFF 423
W5PPU7|CNGB3_O.ARIES        GS-----------TRWVYDGEGNKWYLRCYYWAVRTLITIGGLPEPQTLFEIVFQLLNFF 307
A0A1D5PGA2|CNGB3_G.GALLUS   GS-----------TRWVYDGQGNM-YLRCYYWAVRTLITIGGLPEPQTLFEIVFQLLNFF 372
A0A2R8RTW7|cngb3.2_D.RERIO  GS-----------TKWVYSGKGSA-YLHSYYFAVRTLINIGGLPEPHTIFEISFQLTNFF 450
A0A0R4IL01|cngb3.2_D.RERIO  GS-----------SRWTYDGLGNA-YLRCYYFATRTLITIGGLPEPHTLFEIVFQLVNFF 408
G5EEE2|tax-2_C.ELEGANS      GQIAYLENGKWYLNKWVYNNQGNA-YIRCFYFTAAVATSTGNNPAPTNVIEYIYMTCSWM 442

                        p.(G868D)            p.(A888T)
Q14028|CNGB1_H.SAPIENS      TGVFAFSVMIGQMRDVVGAATAGQTYYRSCMDSTVKYMNFYKIPKSVQNRVKTWYEYTWH 602
Q9NQW8|CNGB3_H.SAPIENS      SGVFVFSSLIGQMRDVIGAATANQNYFRACMDDTIAYMNNYSIPKLVQKRVRTWYEYTWD 488
Q9JJZ9|CNGB3_M.MUSCULUS     SGVFVFSSLIGQMRDVIGAATANQNYFQACMDHIIAYMNKYSIPQSVQYRVRTWLEYTWN 480
Q8MJD7|CNGB3_C.LUPUS        SGVFVFSSLIGQMQDVIGAATANQNNFRISMDHTISYMNTYSIPKNVQNRVRTWYEYTWD 483
W5PPU7|CNGB3_O.ARIES        SGVFVFSSLIGQMQDVIGAATANQNNFRICMDHTIAYMNTYSIPKIVQNRVRTWYEYTWA 367
A0A1D5PGA2|CNGB3_G.GALLUS   LGVFVFSSLIGQMRDVIGAATAGQNYYRSCMDNTVSYMNTYSIPKLVQNRVRTWYEYTWD 432
A0A2R8RTW7|cngb3.2_D.RERIO  VGVFVFSSLIGQMRDVIGAATAGQTYFRASMDACVAYMVTNHIPKMVQSRVRTWYNYTWD 510
A0A0R4IL01|cngb3.2_D.RERIO  TGVFVFSSLIGQMRDVIGAATAGQTYFRASMDSCVAYMNTYTIPKLVQSRVRTWYNYTWD 468
G5EEE2|tax-2_C.ELEGANS      MGVFVFALLLGQIRDIVSNANRNREEFQRKMDLALGECKKLGLKMETTNRVRDWFIYTWQ 502

Q14028|CNGB1_H.SAPIENS      SQGMLDESELMVQLPDKMRLDLAIDVNYNIVSKVALFQGCDRQMIFDMLKRLRSVVYLPN 626
Q9NQW8|CNGB3_H.SAPIENS      SQRMLDESDLLKTLPTTVQLALAIDVNFSIISKVDLFKGCDTQMIYDMLLRLKSVLYLPG 548
Q9JJZ9|CNGB3_M.MUSCULUS     SQRILDESNLLENLPTAMQLSIALDINFSIIDKVELFKGCDTQMIYDLLLRLKSTIYLPG 540
Q8MJD7|CNGB3_C.LUPUS        SQRMLDESDLLCTLPVTMQLALTVDVNLSIISKVELFKGCDTQMIYDMLLRLKSTVYLPG 543
W5PPU7|CNGB3_O.ARIES        SQRMLDESDLLETLPTTMQLALTVDVNFGVISKVDLFKGCDTQMIYDMLLRLKSIVYLPG 427
A0A1D5PGA2|CNGB3_G.GALLUS   SQGMLDESELLEQMPTKMQLAIAIDVNFAIVNKVDLFKGCDTQMIYDMLLRLKSIVYLPG 492
A0A2R8RTW7|cngb3.2_D.RERIO  SQGMLDESELLGQMPLVMRTAIAVDINLATFQKIELFKGCDNQMLVDMLLRLKSIVYLPG 570
A0A0R4IL01|cngb3.2_D.RERIO  SQGMLDESELLDKMPLVMRTAIAVDINLATFQKIDLFKGCDNQMLVDMLLRLKSIVYLPG 528
G5EEE2|tax-2_C.ELEGANS      QQKTLDEKKLIEKLPLKLQTDLALSVHYTTLSKVQLFQDCDRALLRDLVLKLRPVIFLPG 562
```

```
                                  p.(V989E)
Q14028|CNGB1_H.SAPIENS       DYVCKKGEIGREMYIIQAGQVQVLGGPDGKSVLVTLKAGSVFGEISLLA--VGGGNRRTA 684
Q9NQW8|CNGB3_H.SAPIENS       DFVCKKGEIGKEMYIIKHGEVQVLGGPDGTKVLVTLKAGSVFGEISLLA--AGGGNRRTA 606
Q9JJZ9|CNGB3_M.MUSCULUS      DFVCKKGEIGKEMYIIKHGEVQVLGGPDGAQVLVTLKAGSVFGEISLLA--KGGGNRRTA 598
Q8MJD7|CNGB3_C.LUPUS         DFVCKKGEIGKEMYIIKQGEVQVLGGSDGAQVLVTLKAGAVFGEISLLA--GRGGNRRTA 601
W5PPU7|CNGB3_O.ARIES         DFVCKKGEIGKEMYIIKQGEVQVLGGADGTQVLVTLKAGAVFGEISLLA--ARGGNRRTA 485
A0A1D5PGA2|CNGB3_G.GALLUS    DFVCKKGEIGREMYIIKQGEVQVLGGPDGTKVLVTLRAGAVFGEISLLA--AGGGNRRTA 550
A0A2R8RTW7|cngb3.2_D.RERIO   DFVVKKGDIGKEMYIIKAGEVQVIGGPDNKIVFVTLKAGCVFGEISLLQSSANGGNRRTA 630
A0A0R4IL01|cngb3.2_D.RERIO   DFVCKKGDIGREMYIIKAGEVQVIGGPDNKIVFVTLKAGCVFGEISLLQSAKDGGNRRTA 588
G5EEE2|tax-2_C.ELEGANS       DMICLKGDVGKEMYIINQGILQVVGGDHNEKIFAELAQGAVFGEISLLA--IGGNNRRTA 620


Q14028|CNGB1_H.SAPIENS       NVVAHGFTNLFILDKKDLNEILVHYPESQKLLRKKARRMLRSNNK----------PKEEK 734
Q9NQW8|CNGB3_H.SAPIENS       NVVAHGFANLLTLDKKTLQEILVHYPDSERILMKKARVLLKQKAKT-----AEATPPRKD 661
Q9JJZ9|CNGB3_M.MUSCULUS      DVVAHGFANLLTLDKKTLQEILLHYPTSKKLLMKKAKILLSQKGKT-----TQAIPARPG 653
Q8MJD7|CNGB3_C.LUPUS         NVIAHGFANLLTLDKKTLQEILVHYPDSEKLLMKKASVLLKKKAPA-----TETTPPRKG 656
W5PPU7|CNGB3_O.ARIES         NVVAHGFANLLTLDKKTLQEILVHYPDSEKLLMKKARVLLKNKAPG-----TGATPPNKG 540
A0A1D5PGA2|CNGB3_G.GALLUS    NVVAHGFANLFILDKKTLNEILVHYPDSEKLLMKKAKYVLRTIFKSLCLDCKWTVVQPRG 610
A0A2R8RTW7|cngb3.2_D.RERIO   NVAAHGFANLFVLDKKDLNDILIHYPESQKVLARKGRKLLKAKGPPPAAKA---DEEKKK 687
A0A0R4IL01|cngb3.2_D.RERIO   NVAAHGFANLFVLDKKDLNDILIHYPESQKVLAKKGRKLMKAKGKAAPTKD---EGEKKK 645
G5EEE2|tax-2_C.ELEGANS       SIRAKGYCTLFVLAKEDLNDVIRYYPQAQTILRRKAAAMLKNDKKSDEKTEKIKAQAELE 680


Q14028|CNGB1_H.SAPIENS       SVLILPPRAGTPKLFNAALAMTGKMGGKG--------AKGGKLAHLRARLKELAALEAAA 786
Q9NQW8|CNGB3_H.SAPIENS       LALLFPPKEETPKLFKTLLGGTGKASLAR--------LLKLKREQAAQKKENSEGGEEEG 713
Q9JJZ9|CNGB3_M.MUSCULUS      PAFLFPPKEETPRMLKVLLGNTGKVDLGR--------LLKGKRKTTTQK----------- 694
Q8MJD7|CNGB3_C.LUPUS         LAFLFPPKQETPKIFKALLGGTGKAGLTR--------LLKLKREQTIQKTSENS------ 702
W5PPU7|CNGB3_O.ARIES         LAFLFPPKQETPKMFKALLGSSGKAGLAR--------LLTLKREQTGQRKDENQ------ 586
A0A1D5PGA2|CNGB3_G.GALLUS    LASLFGQKQETPKLFKAMFGAKGKGGLAK--------LLQLKREQDIQVMSQIIEQILSR 662
A0A2R8RTW7|cngb3.2_D.RERIO   GLALFGQKPPTPKMLRAFNVVHNISTSH-------------------------------- 715
A0A0R4IL01|cngb3.2_D.RERIO   GLALFGPKPPTPKMLQCYVYTHIRKLHVNASAFASYSLLLSTFERSRVRVRVFTHTLNSN 705
G5EEE2|tax-2_C.ELEGANS       DRCKINPR-QVPKLITLIANMTEMNENKGVQE-----LKKVIE----------EETEKSR 724
```

```
Q14028|CNGB1_H.SAPIENS      KQQELVEQAKSSQD-----VKGEEGSAAPDQHTHPKEAATDPPAPRTPPEPPGSPPSSPP 841
Q9NQW8|CNGB3_H.SAPIENS      KEN--EDKQKENEDKQKENEDKGKENEDKDKGREPEEKPLDRPECTASPIAVEEEP---- 767
Q9JJZ9|CNGB3_M.MUSCULUS     ------------------------------------------------------------
Q8MJD7|CNGB3_C.LUPUS        ------------------EEGGGKRREYEDKEREPSEKILDSSECRANCIIAEEMP---- 740
W5PPU7|CNGB3_O.ARIES        ------------------EEE-DKGKESEDKGRATAEKPLEASKCQTSPITAEEAP---- 623
A0A1D5PGA2|CNGB3_G.GALLUS   ------------------------------------------------------------
A0A2R8RTW7|cngb3.2_D.RERIO  ------------------------------------------------------------
A0A0R4IL01|cngb3.2_D.RERIO  IYNRYF----YSTALH---F---------------------------------------- 718
G5EEE2|tax-2_C.ELEGANS      RQSIYYPWSTLQRD----DDDEEEWNDEEDLSDVGEDFDLDPTNHSDDEDPMEDVDLAPE 780

Q14028|CNGB1_H.SAPIENS      PASLGRPEGEEEGPAEPEEHSVRICMSPGPEPGEQILSVKMPEEREEKAE 891
Q9NQW8|CNGB3_H.SAPIENS      -HSV----RRTVLPRGTSRQSLIISMAPSAEGGEEVLTIEVKEKAKQ--- 809
Q9JJZ9|CNGB3_M.MUSCULUS     --------------------------------------------------
Q8MJD7|CNGB3_C.LUPUS        -QSI----RRAALPRGTTRQSLIISMAPSAEAGEEVLTIEVKEKAKQ--- 782
W5PPU7|CNGB3_O.ARIES        -QAI----RKAVLPRGTTRQSLIISMAPSAEAGEEVLTIEVKEKAKQ--- 665
A0A1D5PGA2|CNGB3_G.GALLUS   --------------------------------------------------
A0A2R8RTW7|cngb3.2_D.RERIO  --------------------------------------------------
A0A0R4IL01|cngb3.2_D.RERIO  --------------------------------------------------
G5EEE2|tax-2_C.ELEGANS      VHDD---DWDQPGTSGT--QKLHAD------------------------- 800
```

Table S3. Conservation analysis of the novel missense variants on *CNGB1,* described in the study. This analysis was performed using the University of California Santa Cruz (UCSC) Genome Browser, available at https://genome.ucsc.edu/index.html. The interested residue is highlighted in red

Nucleotide Genomic position (hg19)	**57953019**	**57912979**	**57938771**	**57938717**	**57938697**	**57938669**	**57937858**	**57935266**
AA position	**647**	**774**	**834**	**852**	**859**	**868**	**888**	**989**
Altered residue	**R**	**K**	**L**	**L**	**I**	**D**	**T**	**E**
H.sapiens	YQFPQSIDPLT	YMAFFEFNSRL	NGSYIRCYYFA	GGLPDPKTLFE	TLFEIVFQLLN	LNYFTGVFAFS	VGAATAGQTYY	LPNDYVCKKGE
P.troglodytes	YQFPQSIDPLT	---	NGSYIRCYYFA	GGLPDPKTLFE	TLFEIVFQLLN	LNYFTGVFAFS	VGAATAGQTYY	LPNDYVCKKGE
M.mulatta	YQFPQSIDPLT	YMAFFEFNNRL	NGSYIRCYYFA	GGLPDPKTLFE	TLFEIVFQLLN	LNYFTGVFAFS	VGAATAGQTYY	LPNDYVCKKGE
R.norvegicus	YQFPQSIDPLT	YMAFFEFNNRL	NGSYIRCYYWA	GGLPDPQTLFE	TLFEIVFQLLN	LNYFTGVFAFS	VGAATAGQTYY	LPNDYVCKKGE
B.taurus	YQFPQSIDPLT	YMAFFEFNNRL	NGSYIRCYYWA	GGLPDPRTLFE	TLFEIVFQGLN	LNYFTGVFAFS	VGAATAGQTYY	LPNDYVCKKGE
C.lupus	YRFPQSIDPLT	YMAFFEFNSRL	NGSYIRCYYWA	GGLPDPRTLFE	TLFEIVFQGLN	LNYFTGVFAFS	VGAATAGQTYY	LPNDYVCKKGE
G.gallus	YQFPSSIDPLT	YLAFFEFNNRL	NGSYIRCYYWA	GGLPDPKTLFE	TLFEIVFQLLN	LNYFTGVFAFS	VGAATAGQTYY	LPNDFVCKKGE
X.tropicalis	LKFPSSIDPLT	YMAFFEFNNRL	NGSYIRCYYWA	GGLPDPDTLFE	TLFELWFQLLN	LNYFMGVFAFS	VGAATAGQTYY	LPGDYVCKKGE
D.rerio	---	YNAFFEFNDRL	NGSYIRCYYFA	GGLPDPTTVFE	TVFEIVFQLVN	VNYFVGVFAFS	IGAATAGEAYY	LPNDFVCEKGE

Table S4. Conservation analysis of the novel canonical and non-canonical splice site variants on *CNGB1,* described in the study. This analysis was performed using the University of California Santa Cruz (UCSC) Genome Browser, available at https://genome.ucsc.edu/index.html. The interested residue is highlighted in red. Caps letters represent exons.

Nucleotide Genomic position (hg19)	58001027	57954451	57953001	57951166	57946900	57911751	57937725	57935346
cDNA Nucleotide position (NM_001297.4)	159+5	1644-3	1957+2	2166+6	2305-2	2492+2	2794+1	2893-7
Altered residue	t	c	c	g	c	c	t	t
H.sapiens	Ccactctccat	agggtgtcACC	TGGTcactcag	cactcagtgtc	ggaggtcATGT	TGTCcactcgg	CGACCcattct	agtggccctgt
P.troglodytes	Ccactctccat	agggtgtcGCC	TGGTcactcag	cactcagtgtc	---	TGTCcacttgg	CGACCcattct	agtggccctgt
M.mulatta	Ccactctccac	agggtgtcGCC	TGGTcactcag	cactcagtgtc	ggaggtcATGT	TGTCcactcgg	CGACCcattct	agtgaccctgt
R.norvegicus	Ccactccccct	ggggtgtcACC	TGGTcactcaa	cattcagtgtc	ggaggtcATGT	TGTCcactcgg	CGACCcattct	gga---cccgt
B.taurus	Ccattcttgac	aaggtgtcACT	TGGTcactcag	cactcagtgtc	ggaggtcATAT	TGTCcactcgg	CGACCcattc-	agagccccggt
C.lupus	Ccattctccga	caggggctACC	TGGTcactcag	cactcagggcc	gaaggtcATGT	TGTCcactcgg	CGACCcattc-	agaacccctgt
G.gallus	---	-gggtgctACG	TGGTcactct-	cactcacctg-	ggaggtcATGG	TGTCca-----	CGATCcactct	aaac--ccggt
X.tropicalis	---	aggacgtcCCA	TGATcattca-	cattcagt---	tacagtcATGT	TGTCcattc--	CGAACcattct	aga----cagt
D.rerio	---	aggacg-----	---	cactcg-----	--a--tcATGT	TGTCcactcg-	CGACCcattc-	---------gt

Table S5. Additional clinical phenotype of the subjects carrying likely disease-causing *CNGB1* variants included in the study**.** No data available for patients SRP480 and SRP629. BCVA: Best corrected visual acuity; OD: right eye; OS: left eye; OU: both eyes; wnl: within normal limits; ERM: epiretinal membrane; SW-FAF: Short-wavelength fundus autofluorescence; OCT: optical coherence tomography; n/a: not applicable.

ID	Age at visit (years)	BCVA (Snellen)		Visual field (degrees) (horizontal axis/vertical axis (isopter))				Color Vision (Axis affected)		SW-FAF		OCT	
		OD	OS	OD	OS	OU (III4)	Other comments	OD	OS	OD	OS	OD	OS
F5240 CIC09166	14	20/50	20/50	140/100 (V4)	140/100 (V4)	140/100	n/a	Tritan		Diffuse hypoautofluorescence in the far periphery with large ring of hyperautofluorescence outside the vascular arcade		Normal	
OPH2286	18	20/32	20/32	130/110 (V4)	130/120 (V4)	145/110	n/a	Not Available		Diffuse peripheral hypoautfluorescence with central area of preserved fluorescence surrounded by ring of hypeaurofluorescence.		Reduction of the retinal thickness outside the center due to atrophy of the outer retinal layers; macular micro-cysts	
SRP541	29	20/25	20/20	Not Available									
ARRP278	30	20/32	20/25	20/20 (III4)	20/20 (III4)	Not available	n/a	Deutan		Diffuse peripheral hypoautofluorescence with central area of preserved fluorescence surrounded by ring of hyperautofluorescence		Reduction of the retinal thickness outside the center due to atrophy of the outer retinal layers, ERM	
TW8999826#	30	20/20	20/20	Not Available						Diffuse peripheral hypoautofluorescence with central area of preserved fluorescence surrounded by ring of hyperautofluorescence		Reduction of the retinal thickness outside the center due to atrophy of the outer retinal layers	
TW20024045#	31	20/20	20/20	Not Available						Diffuse peripheral hypoautofluorescence with central area of preserved fluorescence surrounded by ring of hyperautofluorescence		Reduction of the retinal thickness outside the center due to atrophy of the outer retinal layers	
SRP995	31	20/25	20/20	160/70 (III4) Complete annular	150/70 (III4) Large annular	Not available	n/a	wnl		Diffuse peripheral hypoautofluorescence with central area of preserved fluorescence surrounded by ring of hyperautofluorescence		Reduction of the retinal thickness outside the center due to atrophy of the outer retinal layers	

				scotoma between 20-40° nasally and 10-30° temporally	scotoma between 20-40° nasally and 10-40° temporally					
MRN:6822243	34	20/30	20/30	Not Available					Some hyper-autofluorescent lines in the inferior nasal retina are shown.	Normal
OPH3784	36	20/20	20/25	Not available		145/115	n/a	Not available	Diffuse peripheral hypoautfluorescence with central area of preserved fluorescence surrounded by ring of hypeaurofluorescence. RIF is large, outside the arcades.	Reduction of the retinal thickness outside the center due to atrophy of the outer retinal layers
ARRP400	36	20/20	20/20	120/60 (III4)	130/120 (III4) elliptic area of scotoma between 30° and 40° inferiorly	Not available	n/a	wnl	Diffuse peripheral hypoautofluorescence with central area of preserved fluorescence surrounded by ring of hyperautofluorescence	Reduction of the retinal thickness outside the center due to atrophy of the outer retinal layers, ERM
F4300 CIC07722	39	20/25	20/32	Not Available						
SRP266	41	20/20	20/20	50/40 (III4) Some peripheral islands of	40/40 (III4) Some peripheral islands of	Not available	Some peripheral islands of residual vision in both eyes	wnl	Diffuse peripheral hypoautofluorescence with central area of preserved fluorescence surrounded by ring of hyperautofluorescence	Reduction of the retinal thickness outside the center due to atrophy of the outer retinal layers

				residual vision	residual vision							
ARRP349	41	20/80	20/100	10/10 (III4)	10/5 (III4)	Not available	n/a	Not available		Diffuse peripheral hypoautofluorescence with central area of hyperautofluorescence	Reduction of the retinal thickness outside the center due to atrophy of the outer retinal layers	
F141 CIC00189	43	20/320	20/250	150/110 (V4) Central scotoma	150/110 (V4) Central scotoma	160/110	Central scotoma	Tritan		Central area of hypoautofluorescence, surrounded by annular area of preserved fluorescence. Externally, starting from the main temporal vascular arcades, diffuse grainy peripheral hypoautofluorescence	Diffuse reduction of the retinal thickness due to the atrophy of the inner retinal layers. Diffuse disruption/atrophy of the ellipsoid zone, involving the fovea; intraretinal hyperreflective foci; traces of ERM (not involving the center)	
MEH28189	44	20/30	20/40	Not Available						Diffuse peripheral hypoautfluorescence with central area of preserved fluorescence surrounded by ring of hypeaurofluorescence	Preservation of fovea in both eyes, with good preservation of central EZ	Preservation of fovea in both eyes, with good preservation of central EZ
F1107 CIC01530	45	20/63	20/32	20/20 (V4)	20/20 (V4) Some peripheral islands of residual vision	15/15	Some peripheral islands of residual vision in the left eye	wnl		Diffuse peripheral hypoautofluorescence with central area of preserved fluorescence surrounded by ring of hyperautofluorescence. Focal areas of hyperautofluorescence in the optic disc (drusen of the optic disc)	Central area of preserved retina, surrounded by area of atrophic ellipsoid zone and a more external area of RPE and inner retinal layer atrophy. ERM. Small intraretinal cysts (not involving the center)	
F3791 CIC06919	45	20/20	20/20	150/100 (V4)	150/110 (V4)	160/110	n/a	Tritan		Diffuse peripheral hypoautofluorescence with central area of preserved fluorescence surrounded by ring of hyperautofluorescence	Reduction of the retinal thickness outside the center due to atrophy of the outer retinal layers, ERM	
ARRP386	49	20/20	20/20	20/20 (III4)	20/20 (III4)	Not available	n/a	Not available		Diffuse peripheral hypoautofluorescence with central area of preserved fluorescence surrounded by ring of hyperautofluorescence	Reduction of the retinal thickness outside the center due to atrophy of the outer retinal layers, ERM	
ARRP398	49	20/20	20/20	Not interpretable	40/20 (III4)	Not available	n/a	wnl	Tritan	Diffuse peripheral hypoautofluorescence with central area of preserved	Reduction of the retinal thickness outside the center due to atrophy of the outer retinal layers, ERM	

									fluorescence surrounded by ring of hyperautofluorescence	
OPH1710	50	20/25	20/25	Not Available		145/105	n/a	wnl	Diffuse peripheral hypoautfluorescence with central area of preserved fluorescence surrounded by ring of hypeaurofluorescence. RIF is inside the arcades. Some peripheral punched-out round hypoAF lesions.	Reduction of the retinal thickness outside the center due to atrophy of the outer retinal layers; macular micro-cysts
MRN:8759303	50	20/100	20/30	Not Available						
F463 CIC00691*	51	20/125	20/50	30/20 (V4)	30/20 (V4)	30/20	n/a	Deutan	Diffuse peripheral hypoautofluorescence with central area of preserved fluorescence surrounded by ring of hyperautofluorescence	Central area of preserved retina, surrounded by area of atrophic ellipsoid zone.
F463 CIC02695*	51	20/50	20/20	30/30 (V4)	30/40 (V4)	40/40	n/a	wnl	Diffuse peripheral hypoautofluorescence with central area of preserved fluorescence surrounded by ring of hyperautofluorescence	Central area of preserved retina, surrounded by area of atrophic ellipsoid zone; traces of ERM (not involving the center)
F2070 CIC04317	51	20/20	20/32	20/20 (V4) Some peripheral islands of residual vision	20/20 (V4) Some peripheral islands of residual vision	20/20	Some peripheral islands of residual vision in both eyes	wnl	Diffuse peripheral hypoautofluorescence with central area of preserved fluorescence surrounded by ring of hyperautofluorescence	Central area of preserved retina, surrounded by area of atrophic ellipsoid zone and a more external area (nasally) of RPE and inner retinal layer atrophy.
F5462 CIC09504	52	20/125	20/800	Not available				Deutan	Diffuse peripheral hypoautofluorescence with central area of preserved fluorescence surrounded by ring of hyperautofluorescence	Reduction of the retinal thickness outside the center due to atrophy of the outer retinal layers

<table>
<tr><th></th><th></th><th></th><th></th><th></th><th></th><th></th><th></th><th></th><th colspan="2"></th><th colspan="2"></th></tr>
<tr><td>F4053 CIC07355</td><td>55</td><td>20/25</td><td>20/20</td><td>40/20 (V4)</td><td>40/20 (V4)</td><td>40/20</td><td>n/a</td><td>Tetartan</td><td colspan="2">Diffuse peripheral hypoautofluorescence with central area of preserved fluorescence surrounded by ring of hyperautofluorescence</td><td colspan="2">Central area of preserved retina, surrounded by area of atrophic ellipsoid zone. Intraretinal hyperreflective foci and some intraretinal cysts (not involving the center)</td></tr>
<tr><td>F5830 CIC10130</td><td>56</td><td>20/32</td><td>20/32</td><td>10/10 (V4)</td><td>10/10 (V4)</td><td>15/15</td><td>n/a</td><td>wnl</td><td colspan="2">Diffuse peripheral hypoautofluorescence with central area of preserved fluorescence surrounded by ring of hyperautofluorescence</td><td colspan="2">Central area of preserved retina, surrounded by area of atrophic ellipsoid zone. some intraretinal cysts (not involving the center); traces of ERM in the left eye (not involving the center)</td></tr>
<tr><td>ARRP396</td><td>58</td><td>20/40</td><td>20/63</td><td>5/5 (III4)</td><td>5/5 (III4)</td><td>Not available</td><td>n/a</td><td>Not available</td><td colspan="2">Diffuse peripheral hypoautofluorescence with small central area of preserved fluorescence</td><td colspan="2">Reduction of the retinal thickness outside the center due to atrophy of the outer retinal layers, tubulations, ERM</td></tr>
<tr><td>F3038 CIC05823</td><td>64</td><td>Hands motion</td><td>20/160</td><td>-</td><td>5/5 (V4)</td><td>5/5</td><td>Some residual islands of light perception in the right eye</td><td>Unreliable</td><td colspan="2">Central area of hypoautofluorescence, surrounded by thin annular area of preserved fluorescence. More external diffuse grainy hypoautofluorescence</td><td colspan="2">Diffuse reduction of the retinal thickness due to the atrophy of the inner retinal layers. Diffuse disruption/atrophy of the ellipsoid zone, involving the fovea; intraretinal hyperreflective foci</td></tr>
<tr><td>MRN:1203567687</td><td>72</td><td>No light perception</td><td>No light perception</td><td colspan="5">Not Available</td><td colspan="2">Generalized hypoautofluorescence</td><td colspan="2">Diffuse retinal thinning with outer retinal atrophy</td></tr>
<tr><td>MEH16550</td><td>73</td><td>20/30</td><td>No Light Perception</td><td colspan="5">Not available</td><td>Diffuse peripheral hypoautfluorescence with central area of preserved fluorescence surrounded by ring of hypeaurofluorescence.</td><td>Impossible to perform</td><td>Reduction of the retinal thickness outside the center due to atrophy of the outer retinal layers</td><td>Impossible to perform</td></tr>
</table>

F4517 CIC08096	77	20/10 0	20/12 5	5/5 (V4)	5/5 (V4)	10/1 0	-	Tritan	Diffuse peripheral hypoautofluorescence with small central area of preserved fluorescence	Reduction of the retinal thickness outside the center due to atrophy of the outer retinal layers
SRP480	Not Available									
SRP629	Not Available									

* and # : Subjects are siblings

References

Becirovic E, Nakova K, Hammelmann V, Hennel R, Biel M, Michalakis S. 2010. The retinitis pigmentosa mutation c.3444+1G>A in CNGB1 results in skipping of exon 32. PLoS ONE 5:e8969.

Eisenberger T, Neuhaus C, Khan AO, Decker C, Preising MN, Friedburg C, Bieg A, Gliem M, Charbel Issa P, Holz FG, Baig SM, Hellenbroich Y, et al. 2013. Increasing the yield in targeted next-generation sequencing by implicating CNV analysis, non-coding exons and the overall variant load: the example of retinal dystrophies. PLoS ONE 8:e78496.

Fu Q, Wang F, Wang H, Xu F, Zaneveld JE, Ren H, Keser V, Lopez I, Tuan H-F, Salvo JS, Wang X, Zhao L, et al. 2013. Next-generation sequencing-based molecular diagnosis of a Chinese patient cohort with autosomal recessive retinitis pigmentosa. Invest Ophthalmol Vis Sci 54:4158–4166.

Haer-Wigman L, Zelst-Stams WA van, Pfundt R, Born LI van den, Klaver CC, Verheij JB, Hoyng CB, Breuning MH, Boon CJ, Kievit AJ, Verhoeven VJ, Pott JW, et al. 2017. Diagnostic exome sequencing in 266 Dutch patients with visual impairment. Eur J Hum Genet 25:591–599.

Petersen-Jones SM, Occelli LM, Winkler PA, Lee W, Sparrow JR, Tsukikawa M, Boye SL, Chiodo V, Capasso JE, Becirovic E, Schön C, Seeliger MW, et al. 2018. Patients and animal models of CNGβ1-deficient retinitis pigmentosa support gene augmentation approach. J Clin Invest 128:190–206.

Saqib MAN, Nikopoulos K, Ullah E, Sher Khan F, Iqbal J, Bibi R, Jarral A, Sajid S, Nishiguchi KM, Venturini G, Ansar M, Rivolta C. 2015. Homozygosity mapping reveals novel and known mutations in Pakistani families with inherited retinal dystrophies. Sci Rep 5:9965.

Song J, Smaoui N, Ayyagari R, Stiles D, Benhamed S, MacDonald IM, Daiger SP, Tumminia SJ, Hejtmancik F, Wang X. 2011. High-throughput retina-array for screening 93 genes involved in inherited retinal dystrophy. Invest Ophthalmol Vis Sci 52:9053–9060.

Wang J, Zhang VW, Feng Y, Tian X, Li F-Y, Truong C, Wang G, Chiang P-W, Lewis RA, Wong L-JC. 2014. Dependable and efficient clinical utility of target capture-based deep sequencing in molecular diagnosis of retinitis pigmentosa. Invest Ophthalmol Vis Sci 55:6213–6223.

Xu Y, Guan L, Shen T, Zhang J, Xiao X, Jiang H, Li S, Yang J, Jia X, Yin Y, Guo X, Wang J, et al. 2014. Mutations of 60 known causative genes in 157 families with retinitis pigmentosa based on exome sequencing. Hum Genet 133:1255–1271.
